# Quiescence of adult oligodendrocyte precursor cells requires thyroid hormone and hypoxia to activate Runx1

**DOI:** 10.1038/s41598-017-01023-9

**Published:** 2017-04-21

**Authors:** Yasuhito Tokumoto, Shinpei Tamaki, Yasuaki Kabe, Keiyo Takubo, Makoto Suematsu

**Affiliations:** 1 Department of Biochemistry, Keio University School of Medicine, and Japan Science and Technology Agency (JST), Exploratory Research for Advanced Technology (ERATO), Suematsu Gas Biology Project, 35 Shinanomachi, Shinjyuku-ku, Tokyo 160-8582 Japan; 2 Department of Stem Cell Biology, Research Institute National Center for Global Health and Medicine, 1-21-1 Toyama, Shinjyuku-ku, Tokyo 162-8655 Japan

## Abstract

The adult mammalian central nervous system (CNS) contains a population of slowly dividing oligodendrocyte precursor cells (OPCs), i.e., adult OPCs, which supply new oligodendrocytes throughout the life of animal. While adult OPCs develop from rapidly dividing perinatal OPCs, the mechanisms underlying their quiescence remain unknown. Here, we show that perinatal rodent OPCs cultured with thyroid hormone (TH) under hypoxia become quiescent and acquire adult OPCs-like characteristics. The cyclin-dependent kinase inhibitor p15/INK4b plays crucial roles in the TH-dependent cell cycle deceleration in OPCs under hypoxia. Klf9 is a direct target of TH-dependent signaling. Under hypoxic conditions, hypoxia-inducible factors mediates runt-related transcription factor 1 activity to induce G1 arrest in OPCs through enhancing TH-dependent p15/INK4b expression. As adult OPCs display phenotypes of adult somatic stem cells in the CNS, the current results shed light on environmental requirements for the quiescence of adult somatic stem cells during their development from actively proliferating stem/progenitor cells.

## Introduction

Oligodendrocytes (OLs) are myelinating cells of the vertebrate central nervous system (CNS). They are derived from oligodendrocyte precursor cells (OPCs)^1^, which are also called NG2 glial cells or O-2A cells. In the rat optic nerve, OPCs first appear at the brain-end of the nerve on embryonic day 16 (E16) and migrate to the nerve, reaching the eye-end around the day of birth (E21)^2^. OPCs in the developing rat optic nerve exhibit a limited numbers of cell divisions before they terminally differentiate into OLs: the first OLs appear around birth, and their numbers rapidly increase over the following six weeks until the end of optic nerve myelination^3^. In parallel with this process, rapidly dividing perinatal OPCs disappear from the myelinated nerve, as slowly dividing adult OPCs gradually increase and persist in the adult nerve^4–7^. Whereas less than 5% of OPCs are adult OPCs in the optic nerve on postnatal day 7 (P7), almost 70% of OPCs are adult OPCs by P30^6^. Adult OPCs constitute appoximately 5% of the cells throughout the adult CNS, where they have a crucial role in remyelination following CNS damage througout the life of animal, suggesting that adult OPCs are adult somatic stem cells^8–10^. Fate-mapping studies in transgenic mice have shown that adult OPCs develop from perinatal OPCs^11^. However, the molecular mechanisms underlying the perinatal-to-adult transition remain unknown^12^.

The developmental processes from OPCs into OLs *in vivo* can be reproduced *in vitro* ^13^. OPCs prepared from P7 rat optic nerve differentiate into OLs after up to 8 divisions when cultured in 20% O_2_, either as purified OPCs in serum-free medium containing platelet derived growth factor (PDGF), the major mitogen for OPCs^14, 15^, and thyroid hormone (TH)^16^, or as single OPCs on astrocyte monolayers in 0.5% fetal bovine serum (FBS)^17^. PDGF withdrawal induces premature OPC cell cycle withdrawal and differentiation into OLs^18^, and in the absence of TH, OPCs can continue to proliferate in culture for more than 13 months^19, 20^. During this long-term culture, although the cells acquire adult OPC-like morphologies and cell surface antigens, their cell cycle dose not decelerate^19^.

As oxygen concentration in culture might affect the timing of cell differentiation^21^, we examined the effects of hypoxia on the OL differentiation of perinatal OPCs^22^. Surprisingly, a specific O_2_ concentration of less than 1.5% decelerates the cell cycle of perinatal OPCs without OL differentiation and these cells acquire adult OPC-like characteristics. Furthermore, this deceleration of cell cycle is mediated by runt-related transcription factor 1 (Runx1)^23^ through the promotion of G1 arrest in OPCs via cyclin-dependent kinase inhibitor (CKI) p15/INK4b^24, 25^ induction.

## Results

### Decreasing O_2_ biphasically regulates OPC proliferation in a TH-dependent manner

**Figure 1:**
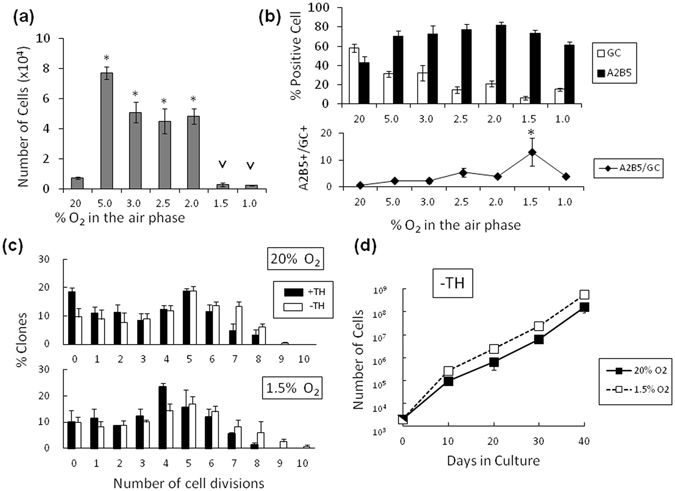
The proliferation and differentiation of purified P7 rat OPCs in clonal-density cultures containing PDGF and TH depends on the O_2_ concentration. (**a**) 2,000 of P7 OPCs were cultured in 1–20% O_2_ for 10 days, and the number of cells was counted. The P value of the cell number compared that of in 20% O_2_ conditions are shown *P < 0.05 (ANOVA with Fisher’s LSD test, n = 3), and compared that of in 2% O_2_ conditions are shown ^V^P < 0.05 (ANOVA, n = 3). (**b**) The cells in (**a**) were stained with anti-GC or A2B5 antibody, and the percentage of GC^+^ cells (*white bars*) and A2B5^+^ cells (*black bars*) was determined (upper panel). And the ratios of A2B5^+^ versus GC^+^ are shown in the lower panel. *P < 0.05 (ANOVA with Fisher’s LSD test, n = 3). (**c**) Clonal analysis of P7 OPCs cultured at clonal-density in PDGF in 20% or 1.5% O_2_ with TH (*black bars*) or without TH (*white bars*). After 11 days, the number of cells in each clone was counted and the number of cell divisions was estimated (n = 3). (**d**) P7 OPCs were cultured in PDGF without TH in either 20% O_2_ (*closed squares*) or 1.5% O_2_ (*open squares*) and were passaged every 10 days (1,000 cells per T25 flask). Cell numbers were estimated from the total cell number at the last passage multiplied by the proliferation rate (n = 3).

Purified P7 rat OPCs (>97% pure) were cultured at clonal density in the serum-free medium containing PDGF and TH^16, 26^. Concentrations of O_2_ in the air interface of the culture ranged between 1% and 20%. The cell numbers after culture for 10 days were significantly greater in 5.0% O_2_ versus 20% O_2_ (Fig. 1a). Between 5.0% and 2.0% of O_2_, the cell numbers modestly decreased. Under conditions with less than 1.5% O_2_, OPCs displayed notably suppressed cell numbers. Immunocytochemistry using the OPC-specific monoclonal antibody A2B5, which recognizes ganglioside GT3^22, 27^, and the monoclonal antibody against galactocerebroside (GC), a cell surface marker of differentiated OLs^28^, revealed that the percentage of GC-positive (GC^+^) cells decreased with decreasing O_2_ level (Fig. 1b, upper panel). Notably, the ratio of the A2B5-positive (A2B5^+^) cells versus the GC^+^ cells reached a maximum at 1.5% O_2_ (Fig. 1b, lower panel), where most of the cells remained A2B5^+^ and GC-negative (GC^−^). The immunocytochemistry result (Supplementary Fig. S1) indicated that the A2B5^+^ cells maintained OPC-specific bipolar bodies in 1.5% O_2_. In OPCs, TH is known to limit the number of cell divisions to execute terminal differentiation into OLs in 20%^16^. Thus we tested whether the effects of TH depend on the O_2_ concentrations by a clonal analysis (see details in Supplementary Fig. S1). In both 20% O_2_ and 1.5% O_2_, the application of TH decreased the number of cell divisions to comparable extents of up to 8 times (Fig. 1c). Conversely, when OPCs were cultured with PDGF in the absence of TH in either 20% or 1.5% O_2_, cells continued to actively proliferate for over 40 days (Fig. 1d), indicating that the decrease in cell number in both 20% and 1.5% O_2_ depends on TH. This also suggests that the lack of increased cell numbers in 1.5% O_2_ does not result from hypoxia *per se*.

As shown in Fig. 1a, OPCs displayed notable proliferation in 3% O_2_. Therefore, we characterized OPC proliferation with 3% O_2_ (Supplementary Fig. S2a). The addition of TH did not inhibit the proliferation of OPCs during the first 10 days. We passaged the cells, re-plated them at clonal density, and cultured them for another 10 days in 3% O_2_. Under these conditions, the cells exhibited cycle withdrawal after up to 7 divisions (Supplementary Fig. S2b); more than 70% of them were GC^+^ OLs (Supplementary Fig. S2c and d). Such phenotypes observed in 3% O_2_ differed from those seen in 1.5% O_2_, and were analogous to those found in 20% O_2_, while the timing of OL differentiation was delayed.

### Hypoxia neither induces cell death and senescence nor inhibits OL differentiation

**Figure 2:**
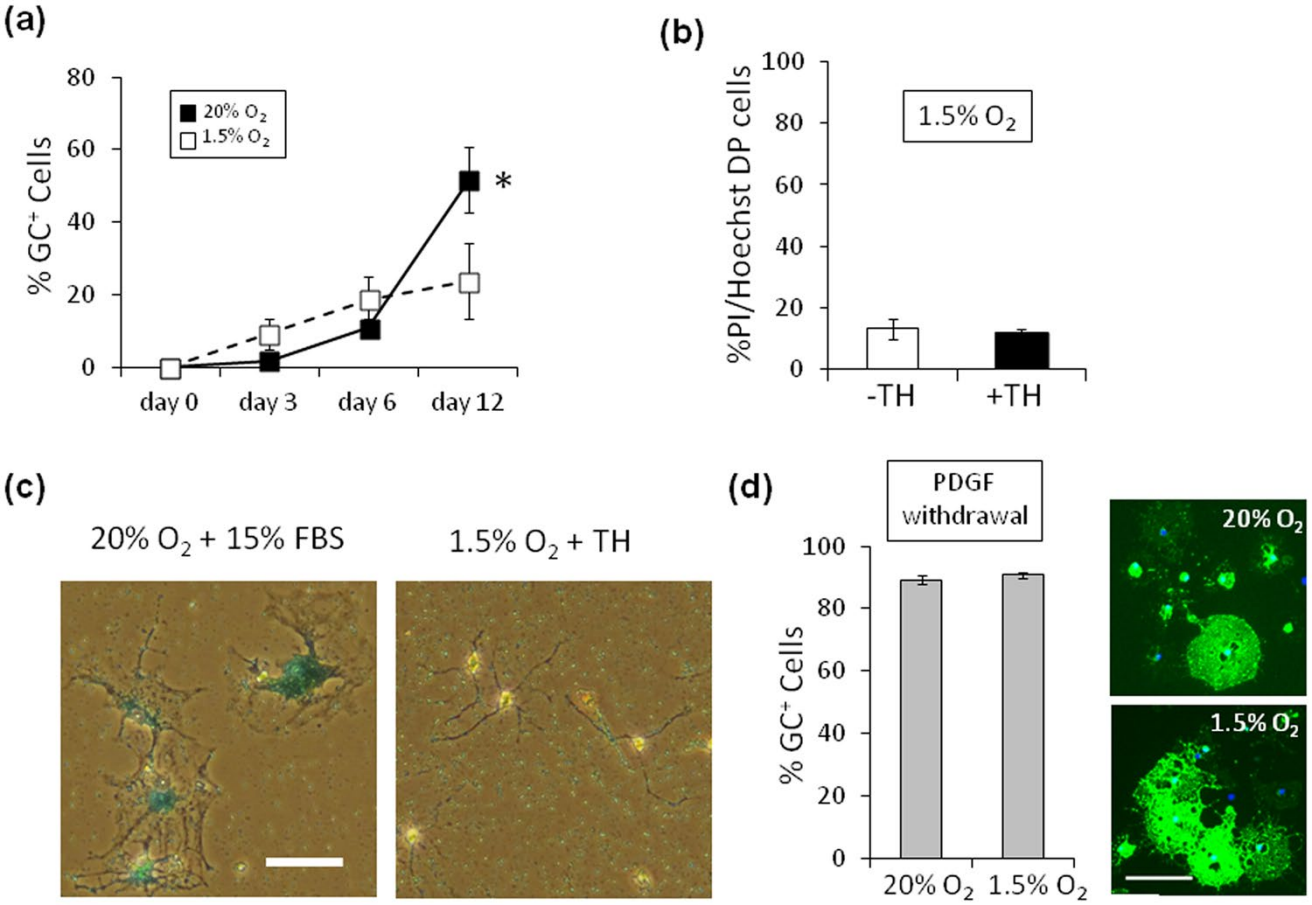
Hypoxia does neither induce cell death and replicative senescence, nor inhibit OL differentiation. (**a**) P7 OPCs cultured in PDGF and TH, in either 20% O_2_ (*closed squares*) or 1.5% (*open squares*), were stained for GC after 3, 6 and 12 days, and the % of GC^+^ cells were counted at each time point. *P < 0.05 (unpaired Student’s *t*-test, n = 3). (**b**) P7 OPCs were cultured in PDGF, without TH (-TH, *white bar*) or with TH (+TH, *black bar*), in 1.5% O_2_ for 15 days, and the percentage of dead cells (PI^+^, Hoechst 33342^+^ double positive cells) was determined (n = 3). (**c**) P7 OPCs were cultured in PDGF and TH in 1.5% O_2_ for 20 days and stained for SA-βGal (left panel); as a positive control, P7 OPCs cultured in 15% FBS in 20% O_2_ for 20 days to induce replicative senescence were also stained for SA-βGal (right panel). Scale bar: 50 μm. (**d**) Freshly purified P7 OPCs were cultured in the absence of PDGF for 3 days, in either 20% O_2_ (upper right panel) or 1.5% O_2_ (lower right panel), and were labeled with anti-GC antibody (*green*) and DAPI (*blue*). Scale bar: 100 μm. Percentages of GC^+^ cells in 20% O_2_ culture and 1.5% O_2_ culture are shown in the graph on the left (n = 3). Note that rat OL differentiation obeying withdrawal of PDGF is not inhibited in 1.5% O_2_, the extensions of GC^+^ membrane-like structure around the cell body in 1.5% O_2_ were comparable with those in 20% O_2_.

In 20% O_2_, the number of GC^+^ OLs was significantly increased during the period between 6 and 12 days, whereas the number of the same cells was not increased during the same period in 1.5% O_2_ (Fig. 2a). Moreover, the failure of increase of OPCs in the presence TH was not because of decreased cell viability, as indicated by only small percentages of dead cells in 1.5% O_2_ (Fig. 2b and Supplementary Fig. S11). Furthermore, the lack of increases in OPCs in 1.5% O_2_ in the presence of TH was not because of the OPCs undergoing replicative senescence^29^: after the 20 days of culture in 1.5% O_2_, the cells retained bipolar OPC morphology without expressing senescence-associated β-galactosidase (SA-βGal)^20, 30^ (Fig. 2c). Although several studies have shown that hypoxia inhibits OL differentiation^31, 32^, hypoxia in 1.5% O_2_ is not sufficient to prevent the terminal differentiation of rat optic nerve OPCs into OLs. As shown in Fig. 2d, PDGF withdrawal caused the rapid differentiation of OPCs into GC^+^ OLs^14, 15, 18^; this event did not differ in 20% O_2_ and 1.5% O_2_.

### Hypoxia converts perinatal OPCs into adult-like OPCs in culture

**Figure 3:**
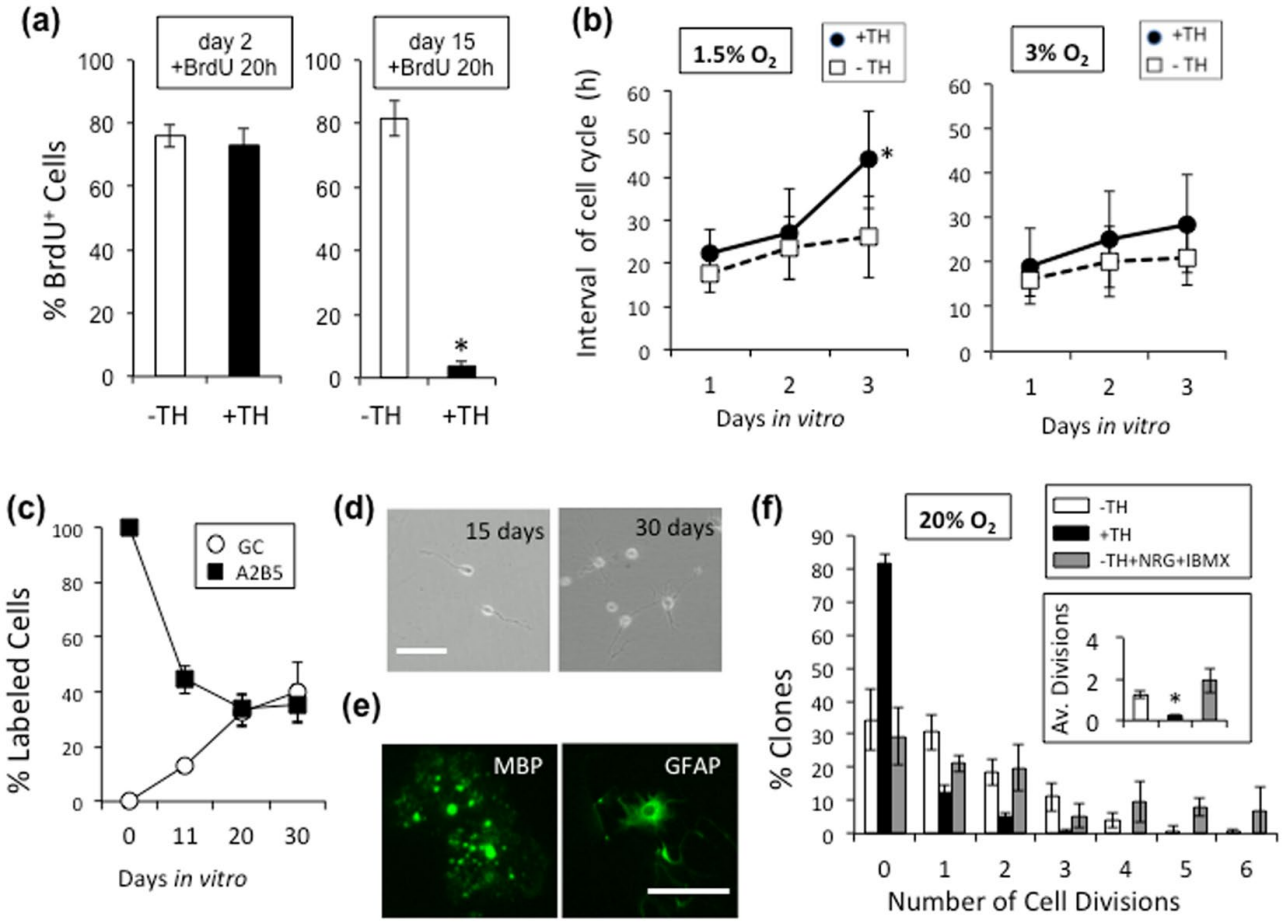
Hypoxia converts perinatal OPCs to adult-like OPCs in culture. (**a**) After 2 or 15 days, with TH (+TH; *black bars*) or without TH (-TH; *white bars*), the P7 OPCs cultured in 1.5% O_2_ condition were treated with BrdU for 20 hours and then the % of BrdU^+^ cells was determined. *P < 0.001. (**b**) Freshly prepared P7 OPCs were plated in PDGF, with TH (*closed circles*) or without TH (*open squares*) and in either 1.5% or 3% O_2_ cultured for 24 hours, after which they were followed by time-lapse video microscopy. The average time between M-phases was estimated on day 1 (0–23.5 hours), day 2 (24–47.5 hours), and day 3 (48–71.5 hours). At the start of image recording, the following number of cells were analyzed: 42 in 1.5% O_2_ with TH; 48 in 1.5% O_2_ without TH; 131 in 3% O_2_ with TH; 89 in 3% O_2_ without TH. *P < 0.05. (**c**) After P7 OPCs were cultured at clonal-density for 11, 20 or 30 days in PDGF, TH, and 1.5% O_2_, the cells were labeled with anti-GC and A2B5 monoclonal antibodies and the percent of each type of labeled cells was determined. (**d**) P7 OPCs were cultured in PDGF and TH in 1.5% O_2_ for 15 or 30 days, and representative fields were examined by phase-contrast microscopy. Scale bar: 50 μm. (**e**) P7 OPCs cultured for 15 days as in (**d**) were then either deprived of PDGF for 5 days and labeled for MBP or treated with 10% FBS for 5 days and labeled for GFAP. Scale bar: 100 μm. (**f**) P7 OPCs cultured in PDGF and TH in 1.5% O_2_ for 15 days were removed and re-cultured at clonal-density for another 7 days in PDGF in 20% O_2_ — without TH (*white bars*), with TH (*black bars*), or without TH in the presence of NRG1 (50 ng/ml) and IBMX (100 μM) (*gray bars*). The average numbers of cell divisions are show in the inset (1.24 ± 0.18 without TH; 0.24 ± 0.60 with TH; 1.95 ± 0.60 without TH, but with NRG1 and IMBX). *P < 0.001.

To examine roles of TH in controlling cell proliferation, freshly prepared P7 OPCs were pre-cultured in the presence of PDGF with or without TH for 2 or 15 days in 1.5% O_2_ and then treated with bromodeoxyuridine (BrdU) for 20 hours. OPCs undergoing the 2-day TH treatment did not suppress the active BrdU incorporation compared with those cultured without TH (Fig. 3a). On the other hand, the 15-day TH treatment suppressed BrdU incorporation, as 3.7 ± 1.2% of the cells were BrdU-positive (BrdU^+^) in the presence of TH, whereas 81.6 ± 5.5% were BrdU^+^ in the absence of TH. We also examined effects of the 40-day TH treatment of P7 OPCs in 1.5% O_2_; the cells were treated with BrdU for the last 5 days. Under these conditions, 1.6 ± 0.8% of the cells were still labeled with BrdU, which was consistent with a notion that adult OPCs exhibit gradually slowed proliferation but a sustained ability to incorporate BrdU *in vivo* ^33^.

We also examined the beginning of the TH-dependent cell cycle deceleration in OPCs. P7 OPCs were cultured with TH in 1.5% or 3% O_2_, and their cell divisions over 5 days were compared using time-lapse microscopy. OPCs treated with TH in 1.5% O_2_, but not those in 3% O_2_, displayed an elongation in the average cell division time, which specifically began on culture day 3 (Fig. 3b). Previous observations have shown that over 95% of P7 rat OPCs cultured in 20% O_2_ became A2B5^−^/GC^+^ OLs within 15 days^34^. We further observed the effects of longer culture periods on the phenotypes of the cells. Treating OPCs cultured in 1.5% O_2_ with TH resulted in a decreased population of A2B5^+^ cells and an increased population of the GC^+^ cells. These two subpopulations reached a plateau around at day 20 (Fig. 3c). Collectively, in the presence of TH, the hypoxic culture helps sustain undifferentiated A2B5^+^ cells for longer period.

Over time, the A2B5^+^ cells exhibiting cell cycle deceleration in 1.5% O_2_ acquired multi-process morphologies resembling that of adult OPCs^4–7^ (Fig. 3d). These multi-process cells moved slowly; according to an analysis using time-lapse video microscopy, when P7 OPCs were cultured in 1.5% O_2_ for 21 days, the bipolar cells not treated with TH were highly motile and proliferated rapidly (Video 1). On the other hand, the multi-process cells treated with TH exhibiting cell cycle deceleration were much less motile (Video 2); this slow movement demonstrated by the cells was similar to that shown by adult OPCs *in vivo* ^35^. And these multi-process cells were could be maintained in 1.5% O_2_ with TH for more than 4 months without differentiating into OLs.

Similar to perinatal OPCs^22^, adult OPCs are induced to differentiate either into OLs by the withdrawal of PDGF or into type-2 astrocytes by the addition of 10% FBS^4, 5, 7^. As shown in Fig. 3e, when P7 OPCs which were cultured in 1.5% O_2_ with PDGF and TH for 15 days and then in PDGF-free medium for 5 days, they acquired morphological features of OLs and expressed OL marker, myelin basic protein (MBP). On the other hand, P7 OPCs, which were treated for 15 days with PDGF and TH, followed by a 5-day treatment with 10% FBS, the cells exhibited the characteristic morphology of type-2 astrocytes^22^ and expressed glial fibrillary acidic protein (GFAP). In addition, using a time-lapse video microscopy, we confirmed the morphological changes from OPCs into OLs (Video 3) and into type-2 astrocytes (Video 4) under hypoxia. These data indicate that the cells with a decelerated cell cycle under hypoxia maintained the developmental bipotency of OPCs.

Adult OPCs isolated from adult rat optic nerve can be stimulated to proliferate rapidly by a combination of PDGF, neuregulin 1 (NRG1), and isobutyl methylxanthine (IBMX) in TH-free culture^7^. To test if this is also the case for the cell cycle-decelerated cells under hypoxia, we dissociated the cells after 15 days of hypoxic culture with TH and re-plated them at clonal density in 20% O_2_, with or without TH, in the presence of PDGF, NRG1, and IBMX for 7 days (Fig. 3f). The cells cultured without TH in the presence of NRG plus IBMX divided rapidly up to 6 times without differentiation.

Taken together, the phenotypes of P7 rat optic nerve OPCs treated with PDGF and TH for 15 days in 1.5% O_2_ *in vitro* are consistent with those of adult OPCs prepared from adult rat optic nerve^5, 7^. Based on these findings, perinatal OPCs cultured with PDGF and TH under hypoxia for over two weeks are characterized by slow proliferation and an A2B5^+^ phenotype with developmental bipotency, and thus are designated adult-like OPCs.

### p15/INK4b induces G1 arrest in adult-like OPCs

**Figure 4:**
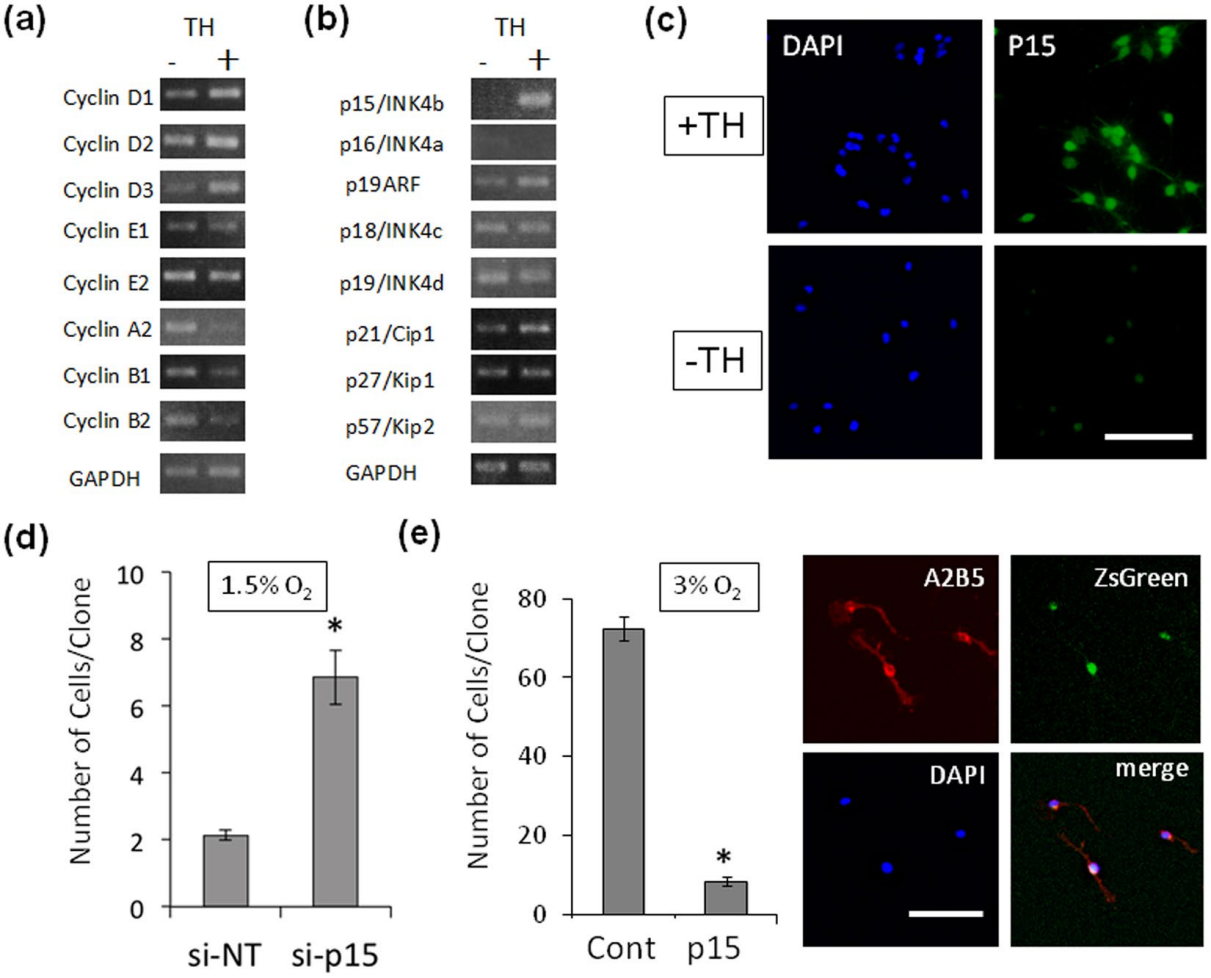
An increase in p15/INK4b in hypoxic OPCs in culture helps mediate the TH-dependent G1 arrest of OPCs. (**a** and **b**) P7 OPCs were cultured in 1.5% O_2_ in PDGF, with or without TH, for 15 days, and assayed by RT-PCR. GAPDH was used as an internal control. (**a**) In Cyclin genes, TH decreased the mRNA for G2/M Cyclins A2, B1 and B2. (**b**) In CKI genes, TH strongly increased the mRNA for p15/INK4b. (**c**) P7 OPCs were cultured in PDGF without TH for 10 days in 1.5% O_2_, and then TH was added to some cultures (+TH) but not to others (−TH) for 2 days. The cells were then labeled with rabbit anti-p15/INK4b antibodies (*green*) and DAPI (*blue*). Note that p15/INK4b protein increased in the nucleus of the TH-treated cells. Scale bar: 100 μm. (**d**) P7 OPCs cultured in 3% O_2_ in PDGF without TH for 12 days were co-transfected with anti-p15/INK4b siRNA (si-p15) and GFP expressing transfection-reporter plasmid DNA (pMaxGFP); a non-targeting siRNA pool (si-NT) served as a negative control. Cells were re-cultured at clonal-density in 1.5% O_2_, PDGF and TH for another 5 days. The number of GFP^+^ cells in each clone was counted. Note that the anti-p15/INK4b siRNA prevented the deceleration of cell cycle that occurs in 1.5% O_2_ in the presence of TH. *P < 0.001 (unpaired Student’s *t*-test, n = 3). (**e**) Freshly prepared P7 OPCs were infected with either a p15/INK4b-IRES-ZsGreen expressing retrovirus vector (p15) or the ZsGreen expressing empty vector as a negative control (Cont), and the cells were cultured at clonal-density in 3% O_2_ and PDGF without TH for 7 days. The number of ZsGreen^+^ cells in each clone was counted, and the results are shown in the graph on left. Note that the cells over expressing p15/INK4b proliferated much less than control. *P < 0.001 (unpaired Student’s *t*-test, n = 3). The p15/INK4b-IRES-ZsGreen expressing cells were also labeled with A2B5 antibody (*red*) and DAPI (*blue*); as shown in the panels on right, the ZsGreen^+^ OPCs (*green*) are bipolar and A2B5^+^. Scale bar: 100 μm.

To investigate mechanisms for the TH-dependent deceleration of the cell cycle in OPCs, total RNA was extracted from P7 OPCs cultured in 1.5% O_2_ with or without TH for 15 days, and gene expressions were analyzed quantitatively on microarray (Supplementary Table S1 **)**. Among 129 of the TH-dependent up-regulated genes, we identified the gene of p15/INK4b (*Cdkn2b*)^24^. Among 60 of the TH-dependent down-regulated genes, 48 genes (80%), including Cyclin A2 (*Ccna2*), Cyclin B1 (*Ccnb1*) and Cyclin B2 (*Ccnb2*), were related to the cell cycle progression. Subsequently, we confirmed the TH-dependent gene expressions of Cyclins and CKIs. RT-PCR analysis showed that TH modestly increased the mRNA levels of G1 Cyclins, Cyclin D1 and D3, and decreased the mRNA levels of G2/M Cyclins, Cyclin A2, B1 and B2 (Fig. 4a). These data suggest that the TH-dependent deceleration of cell cycle in adult-like OPCs results from G1 arrest. p15/INK4b protein induces G1 arrest via direct binding to Cdk4^24^. TH notably increased the mRNA level of p15/INK4b, with negligible effects on mRNA levels of other CKIs tested (Fig. 4b). TH also increased the p15/INK4b protein level in OPCs in 1.5% O_2_ (immunocytochemistry, in Fig. 4c; western blotting, in Supplementary Fig. S3b).

To examine effects of down- or up-regulation of p15/INK4b gene expression on proliferation of adult-like OPCs, we cultured P7 OPCs without TH for 12 days in 1.5% O_2_ and then transfected them with anti-p15/INK4b siRNA. The siRNA inhibited the cell cycle deceleration in perinatal OPCs induced by TH in 1.5% O_2_ (Fig. 4d). Conversely, when a retroviral vector was used to overexpress p15/INK4b in freshly isolated P7 OPCs, which were then cultured for 7 days at clonal-density without TH in 3% O_2_, where adult-like OPCs are never seen, the number of cell division was reduced and the cells maintained the A2B5^+^ bipolar morphology (Fig. 4e). These results suggest that p15/INK4b is an effector molecule that mediates the TH-dependent cell cycle deceleration in adult-like OPCs cultured in 1.5% O_2_.

### Klf9 is the major mediator of TH signal to induce adult-like OPCs

**Figure 5:**
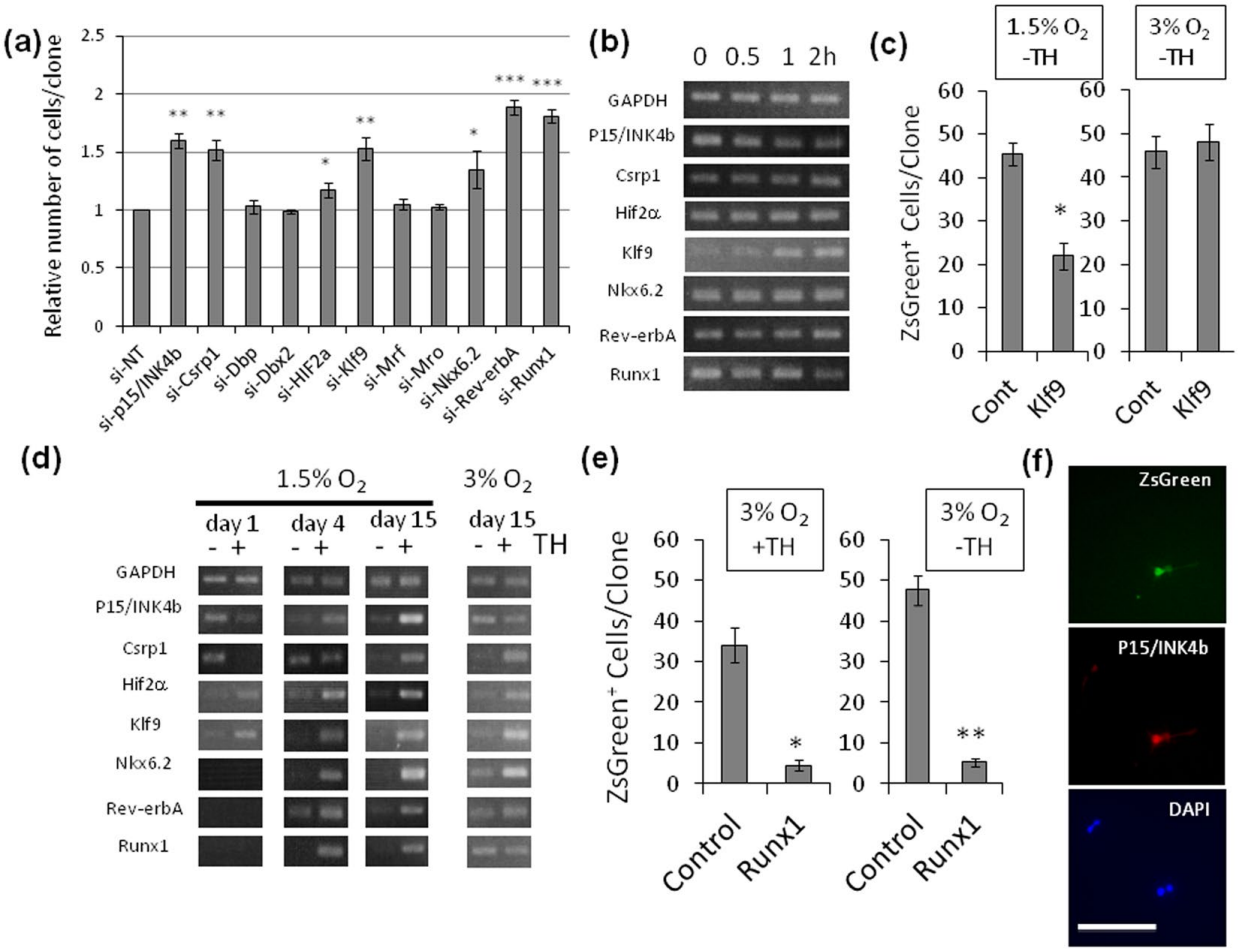
*Runx1* dictates the cell cycle deceleration of OPCs in hypoxia. (**a**) P7 rat OPCs were cultured without TH in 1.5% O_2_ conditions for 12 days, then the cells were co-transfected with anti-p15/INK4b siRNA (si-p15/INK4b) or siRNA against the gene of each transcription factor and pMaxGFP. Cells were cultured with TH in 1.5% O_2_ conditions for another 4 days. The number of GFP^+^ cells in each clone was counted. Data was normalized against the average number of negative control (cells transfected with non-target siRNA; si-NT). *P < 0.05, **P < 0.01, ***P < 0.001 (unpaired Student’s *t*-test, n = 3). (**b**) To investigate the immediate early responses of the genes of the deceleration of cell cycle related transcription factors, P7 rat OPCs cultured without TH for 10 days in 1.5% O_2_ were pre-treated with cycloheximide (50 μg/ml) for 6 hours, after then TH was added to the medium. Cells were harvested at 0, 0.5, 1 and 2 hours later. RT-PCR assay was carried out. GAPDH was used as a negative control. (**c**) P7 rat OPCs were infected with retrovirus vector Klf9-IRES-ZsGreen and were cultured for 7 days without TH in 1.5% or 3% O_2_ conditions. Cont; empty vector RetroX-IRES-ZsGreen1 infected OPCs, Klf9; Klf9 over expressing cells. *P < 0.001 (unpaired Student’s *t*-test, n = 3). (**d**) P7 rat OPCs cultured with (+) or without TH (−) in 1.5% O_2_ for 1, 4 and 15 days, or in 3% O_2_ for 15 days. Then RT-PCR assay was carried out. GAPDH was used as an internal control. (**e**) Freshly prepared P7 OPCs were cultured without TH in 3% O_2_ conditions overnight. Then cells were infected with Runx1 (Runx1b-IRES-ZsGreen) over-expressing retrovirus vector (Runx1) or ZsGreen expressing empty vector (Control). Cells were cultured for 7 days with or without TH in 3% O_2_. The number of ZsGreen^+^ cells in each clone was counted. *P < 0.005, **P < 0.001 (unpaired Student’s *t*-test, n = 3). (**f**) OPCs overexpressing Runx1 (*green*) in (d) were stained with the nuclear stain DAPI (*blue*) and rabbit anti-p15/INK4b antibodies (*red*). Scale bar: 100 μm.

As the expression of p15/INK4b in OPCs was regulated transcriptionally, the mechanisms by which TH induces p15/INK4b were further examined. The microarray data in Supplementary Table S1 shows that of the genes encoding transcription factors, 10 of them (Csrp1, Dbp, Dbx2, Hif2α, Klf9, Mrf, Mro, Nkx6.2, Rev-erbA and Runx1) are transcriptionally up-regulated with TH, and 5 of them (Cbx2, Csrp2, Foxm1, Klf14 and Mybl2) are down-regulated with TH. Individual genes of transcription factors up-regulated with TH were assessed by siRNA-mediated gene silencing to examine the effects on OPC proliferation. The gene silencing of 6 transcription factors (Csrp1, Hif2α, Klf9, Nkx6.2, Rev-erbA and Runx1) inhibited the TH-dependent cell cycle deceleration in OPCs in 1.5% O_2_ (Fig. 5a). The efficiencies of siRNAs were confirmed by western blotting (Supplementary Fig. S4a).

To determine whether the gene expression levels of these transcription factors are under the direct control of thyroid hormone receptor alpha 1^36^, an immediate early-response assay was performed^37^. P7 rat OPCs cultured without TH in 1.5% O_2_ for 10 days were pre-treated with cycloheximide for 6 hours, after which TH was added to the medium. The alterations in the gene expression levels of the transcription factors (Csrp1, Hif2α, Klf9, Nkx6.2, Rev-erbA and Runx1) and p15/INK4b were assessed by RT-PCR as a function of time after the TH treatment. Klf9 (*Klf9*) was the only one gene showing an immediate early-response to the TH stimulation (Fig. 5b). Klf9 (Krüeppel-like factor 9) is a GC-box binding transcription factor that has been considered essential for the TH-dependent differentiation into OLs in cultures with 20% O_2_^38^. Therefore, we examined the effects of Klf9 overexpression in P7 OPCs cultured without TH in 1.5% or 3% O_2_ conditions for 7 days. In the absence of TH in 1.5% O_2_, the overexpression of Klf9 induced the cell cycle deceleration in OPCs (Fig. 5c). However, in 3% O_2_, no cell cycle deceleration in Klf9 overexpressing OPC was seen. Note that, in both O_2_ conditions, Klf9 overexpression did not accelerate OL differentiation, as the cells maintained OPC-specific A2B5^+^ bipolar bodies (Supplementary Fig. S5 and Supplementary Fig. S6a). Collectively, although Klf9 plays a crucial role in TH-signaling in adult-like OPCs, other factors that are induced with hypoxia must be needed for the cell cycle deceleration in OPCs.

### Runx1 determines OPC cell cycle deceleration

To determine which transcription factor(s) responsible for the deceleration of cell cycle show the 1.5% O_2_ condition specific gene up-regulation, we then prepared total RNA from P7 rat OPCs cultured with or without TH in 1.5% O_2_ for 1, 4 and 15 days, as well as from the same cells cultured in 3% O_2_ for 15 days. The gene expression patterns of the six transcription factors were compared with those of p15/INK4b by RT-PCR (Fig. 5d **)**. TH-dependent gene up-regulation of p15/INK4b was undetectable on day 1, showed a modest elevation on day 4, and was finally evident on day 15 in 1.5% O_2_, whereas the TH-dependent up-regulation of the p15/INK4b gene was undetectable on day 15 in 3% O_2_ conditions. These results support the idea that p15/INK4b is the major effector molecule of the TH-dependent cell cycle deceleration in OPCs in 1.5% O_2_. Among six transcription factors, Runx1 was the only one that showed the same TH-dependent gene expression pattern as p15/INK4b.

Thus, we hypothesized that the transcription factor Runx1 ultimately determines the TH-dependent cell cycle deceleration in OPCs in 1.5% O_2_. In rodents, the gene expression of Runx1 is controlled by the dual promoters P1 and P2; these promoters generate two major protein variants (Runx1c and Runx1b)^39^. In hypoxic OPCs, TH activates the proximal promoter P2 and induces the expression of the Runx1b variant (Supplementary Fig. S7). Freshly prepared P7 rat OPCs were transfected with a retroviral vector for Runx1 (variant Runx1b) overexpression and cultured with or without TH in 3% O_2_ for 7 days. Notably, Runx1 over-expression decelerated the cell cycle of OPCs irrespective of the presence or absence of TH or hypoxia (Fig. 5e). The cells maintained OPC-specific A2B5^+^ bipolar morphologies (Supplementary Fig. S6b). These results suggested that the gene expression of Runx1 is crucial for the TH-dependent cell cycle deceleration in OPCs in 1.5% O_2_. Runx1 is known to determine the expression of p15/INK4b^40^. Runx1 overexpression increased p15/INK4b protein level in OPCs in 3% O_2_ (Fig. 5f). These results suggest that the timing of the Runx1 gene expression determines the timing of the TH-dependent cell cycle deceleration in OPCs in 1.5% O_2_.

### Hypoxia-inducible factors induce Runx1 gene expression

**Figure 6:**
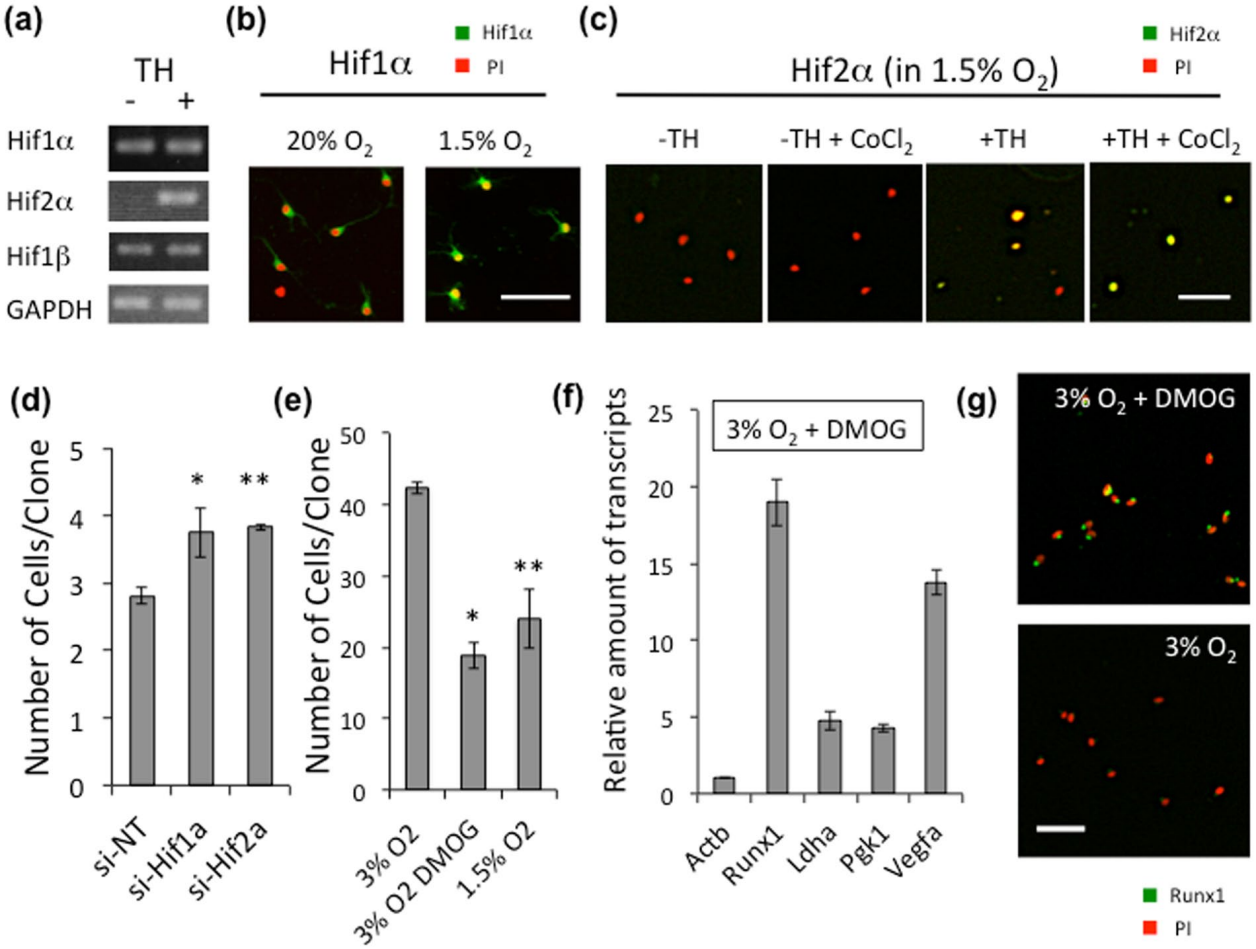
Stabilization of Hifα proteins permits TH-dependent Runx1 gene expression. (**a**) P7 OPCs cultured in 1.5% O_2_ in PDGF, with (+) or without TH (−), for 15 days were assayed gene expressions of Hifs by RT-PCR. (**b**) P7 OPCs were cultured in 20% O_2_ in PDGF without TH for 7 days, and some were then changed to 1.5% O_2_ for 20 hours. The cells were labeled with rabbit anti-Hif1α antibodies (*green*) and PI (*red*). Scale bar: 50 μm. (**c**) P7 OPCs were cultured in 1.5% O_2_ in PDGF with (+TH) or without TH (−TH) for 10 days. The cells were labeled with rabbit anti-Hif2α antibodies (*green*) and PI (*red*). To prevent the degradation of Hif2α protein, some cultures were treated with 0.2 mM of CoCl_2_^65^ for last 7 hours. Scale bar: 50 μm. (**d**) P7 rat OPCs were cultured without TH in 1.5% O_2_ conditions for 12 days, then co-transfected with anti-Hif1α siRNA or anti-Hif2α siRNA and pMaxGFP. Cells were cultured with TH in 1.5% O_2_ conditions for another 4 days. The number of GFP^+^ cells in each clone was counted. si-NT; non-target siRNA, si-Hif1a; anti-Hif1α siRNA, si-Hif2a; anti-Hif2α siRNA. *P < 0.05, **P < 0.01. (**e**) Freshly prepared OPCs from P7 rat optic nerve were cultured with TH in 1.5% O_2_ or 3% O_2_ conditions. For several flasks in 3% O_2_, DMOG (1 mM) was added. After 7 days, the number of cells in each clone was counted. *P < 0.005, **P < 0.05. (**f**) P7 rat OPCs cultured in 3% O_2_ with TH were treated with DMOG (1 mM) for 24 hours. Then qRT-PCR analysis was carried out. Results were presented as the relative amount of transcripts to that of the DMOG free culture using comparative ∆∆Ct method. *Actb* is an endogenous negative control. *Ldha*, *Pgk1* and *Vegfa* are HIFs-inducible positive control. The P values of these genes are P < 0.001 (ANOVA with Fisher’s LSD test, n = 3). (**g**) After 24 hours of DMOG treatment, OPCs (3% O_2_ + TH) were stained with rabbit anti-Runx1 antibodies (*green*) and PI (*red*). Scale bars; 50 μm.

We attempted to investigate the mechanism underlying the hypoxia-dependent Runx1 gene expression. Among the six TH-dependent up-regulated transcription factors, Hif2α (*Epas1*) is an α-subunit of the hetero dimeric transcription factor known as hypoxia-inducible factors (HIFs)^41^. Other subunits of HIFs, Hif1α (another α-subunit of HIFs) and Hif1β (common β-subunit of HIFs) are constitutively expressed in OPCs (Fig. 6a). In hyperoxic conditions, Hifα proteins (Hif1α or Hif2α) are hydroxylated by prolyl-hydroxylase (PHD) and degraded by proteasome. In 1.5% O_2_, both Hif1α and Hif2α proteins are stabilized and accumulated in nucleus of OPCs (Fig. 6b,c). The administration of chetomin^42^ at 2 μM or CAY10585^43^ at 10 μM, both of which can inhibit Hif1α and Hif2α, induced the cell death of OPCs in 1.5% O_2_ (Supplementary Fig. S8). These results suggest that HIFs are necessary for the survival of OPCs under hypoxia. On the other hand, the siRNA mediated gene silencing of Hif1α or Hif2α showed that inhibiting both of them inhibited the TH-dependent cell cycle deceleration in OPCs in 1.5% O_2_ (Fig. 6d). Conversely, the stabilization of Hifα proteins decelerated the cell cycle in OPCs. The administration of a PHD inhibitor N-(2-methoxy-2-oxoacetyl)glycine methyl ester (DMOG)^44^, which stabilizes Hifα proteins, to OPCs cultured with TH in 3% O_2_ induced the cell cycle deceleration to an extent comparable to that observed in 1.5% O_2_ (Fig. 6e). Furthermore, DMOG increased Runx1 gene expression in OPCs cultured with TH in 3% O_2_, in parallel with the up-regulation of HIFs-related genes^41^, such as lactate dehydrogenase A (*Ldha*), phosphoglycerate kinase 1 (*Pgk1*) and vascular endothelial growth factor A (*Vegfa*) (Fig. 6f). Consequently, Runx1 protein level was increased in OPCs treated with TH and DMOG in 3% O_2_ (Fig. 6g), suggesting that the Runx1 gene expression is under the control of transcription factor HIFs, and that the O_2_ sensing of OPCs mainly depends on the stabilities of Hifα proteins.

### Relevance of adult-like OPCs in culture to adult OPCs in rat optic nerves

**Figure 7:**
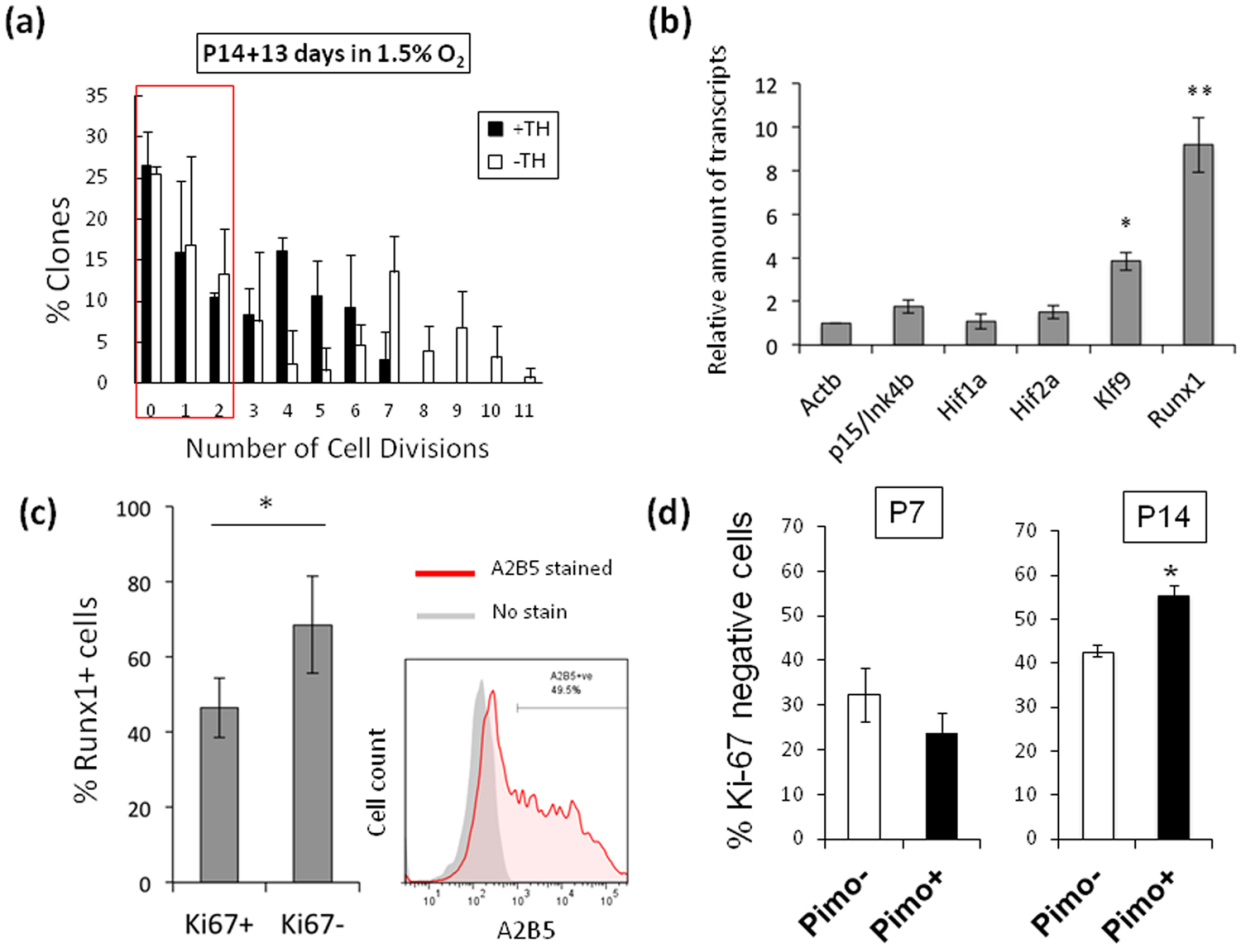
Relevance of adult-like OPCs in culture and adult OPCs in P14 rat optic nerves. (**a**) Clonal analysis of P14 A2B5^+^/GC^−^ OPCs cultured at clonal-density in PDGF in 1.5% O_2_ with TH (*black bars*) or without TH (*white bars*). After 13 days, the number of cells in each clone was counted and the number of cell divisions was estimated (n = 3). Around 50% of clones contained the cells of which divided less than two times are shown in red square. (**b**) P7 OPCs and P14 OPCs were purified from rat optic nerve and their total RNA were prepared immediately. qRT-PCR analysis was carried out. The resulting values were normalized to the endogenous control gene, *Actb*. Results of P14 OPCs are presented as the relative expression to those of P7 OPCS using the ∆∆Ct methods. *P < 0.001, **P < 0.001 (ANOVA with Fisher’s LSD test, n = 3). (**c**) GC-negative optic nerve cells prepared from P14 rat were sorted with A2B5 monoclonal antibody (*right panel*). These GC^−^/A2B5^+^ were stained for Runx1 and Ki-67 and examined in immunochemistry. The percentages of Runx1 expressing cells in Ki-67^+^ or Ki-67^−^ cells are shown (*left graph*). *P < 0.01 (unpaired Student’s *t*-test, n = 10). (**d**) Pimonidazole was injected intraperitoneally into P7 rats or P14 rats. 2 hours later, OPCs were purified from the rat optic nerves. These cells were stained for pimonidazole and Ki-67. The percentages of Ki-67^−^ OPCs that were Pimo^−^ (*white bars*) or Pimo^+^ (*black bars*) at P7 and P14 are shown. *P < 0.001 (unpaired Student’s *t*-test, n = 4).

To examine the relevance of the adult-like OPCs in culture and adult OPCs *in vivo*, we then compared the gene expression level of the adult-like OPC-related transcription factors in freshly prepared A2B5^+^/GC^−^ optic nerve OPCs from P7 rat and in those from P14 rat. Previous studies have shown that less than 5% of OPCs in P7 rat optic nerves are adult OPCs^6^ and more than 50% of OPCs in P14 rat optic nerves are adult OPCs^45^. A clonal analysis showed that approximately 50% of purified A2B5^+^/GC^−^ OPCs in P14 rat optic nerves divided fewer than 3 times over 13 days of culture in 1.5% O_2_, regardless of the presence or absence of TH (Fig. 7a). This finding suggests that these slowly dividing P14 OPCs are committed to becoming adult OPCs. The results of qRT-PCR showed that Runx1 and Klf9 mRNA levels were more than 9-fold and 4-fold greater, respectively, in P14 OPCs than those in P7 OPC (Fig. 7b). Furthermore, we attempted to confirm the increase in the Runx1 protein level in slowly dividing P14 OPCs; FACS sorted A2B5^+^/GC^−^ P14 OPCs were stained with anti-Runx1 and anti-Ki67 antibodies. Ki67 is a protein marker of proliferating cells; thus, we considered Ki-67-negative (Ki-67^−^) OPCs as adult OPCs. The percentage of Ki67^−^ cells expressing Runx1 protein was significantly greater than that of Ki67^+^ cells (Fig. 7c), suggesting that Runx1 contributes to the development of adult OPCs *in vivo*.

To explore OPCs under hypoxic conditions in the developing rat optic nerve *in vivo*, we used pimonidazole, which is activated in hypoxic cells to form stable covalent adducts with thiol groups in proteins^46^. We injected pimonidazole intraperitoneally into both P7 and P14 rats and left the animals for 2 hours. Then, we purified the OPCs from their optic nerves and stained the cells for pimonidazole and Ki-67. As shown in Supplementary Fig. S9b, in both P7 and P14 OPCs, about one-third of the OPCs were pimonidazole-positive (Pimo^+^), suggesting that these cells were exposed to hypoxic environments *in vivo*. At P7 when there are relatively few adult OPCs, only 23.7 ± 4.6% of the Pimo^+^ cells were Ki-67^−^ (Fig. 7d). On the other hand, at P14, 55.4 ± 2.3% of the Pimo^+^ cells were Ki-67^−^. These results suggest that hypoxic conditions in the developing rat optic nerve promote the transition of perinatal OPCs to adult OPCs.

### TH-dependent cell cycle deceleration in rat cortex OPCs and mouse OPCs

**Figure 8:**
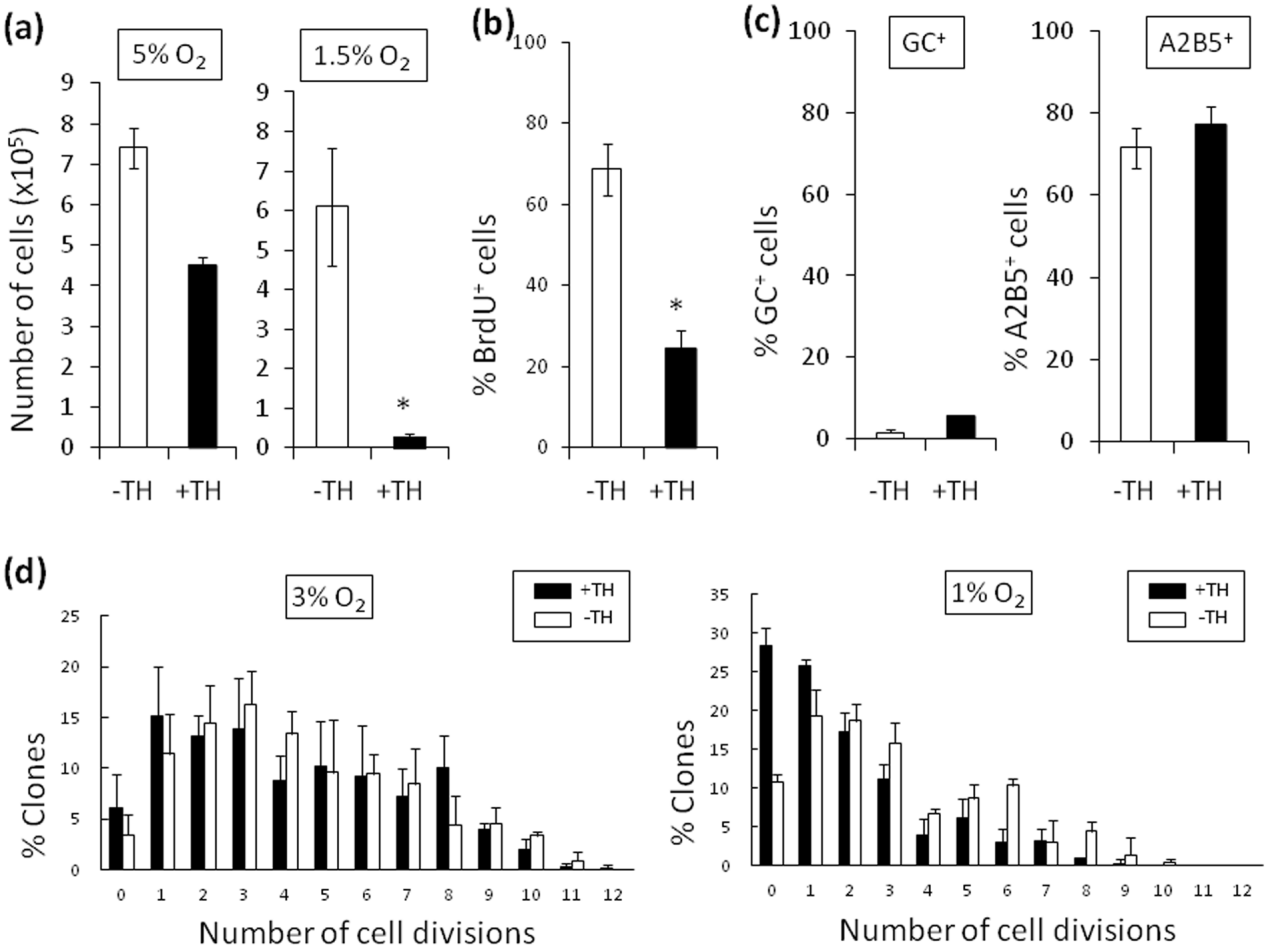
TH-dependent deceleration of cell cycle of OPCs purified from P7 rat cerebral cortex or P7 mouse optic nerve. Purification of OPCs from P7 rat cerebral cortex was carried out obeying Dugas *et al*.^47^. 2,000 of OPCs were cultured without TH (−TH; *white bars*) or with TH (+TH; *black bars*). (**a**) Cells were cultured in 5% O_2_ or 1.5% O_2_. The numbers of cells were counted at day 11. 0.01 < *P < 0.02 (unpaired Student’s *t*-test, n = 3). (**b**) Cells cultured in 1.5% O_2_ for 10 days were treated with BrdU for 20 hours and then labeled with anti-BrdU antibody and Hoechst 33342 dye, and the percentage of BrdU^+^ cells was determined. *P < 0.01 (unpaired Student’s *t*-test, n = 3). (**c**) Cells were cultured in 1.5% O_2_ for 11 days. They were then stained with the nuclear stain DAPI and a monoclonal anti-GC or A2B5 antibody, and the percentage of the DAPI^+^ cells that were GC^+^ or A2B5^+^ was determined (unpaired Student’s *t*-test, n = 3). Note that most of the cells cultured were remained OPC specific morphologies with GC^−^, A2B5^+^. (**d**) OPCs were purified from P7 C57BL/6 mouse optic nerve by immunopanning according to Watkins *et al*.^48^ and cultured at clonal-density according to Durand *et al*.^64^. The cells were cultured for 12 days in PDGF, with TH (*black bars*) or without TH (*white bars*), in either 3% or 1% O_2_. The number of cell divisions was estimated from the cell numbers in each clone (n = 3).

OPCs purified from P7 rat cerebral cortex^47^ clearly showed TH-dependent cell cycle deceleration in 1.5% O_2_ (Fig. 8a–c). Moreover, OPCs purified from P7 mouse optic nerves^48^ also showed the TH-dependent cell cycle deceleration in hypoxic culture, while the hypoxic conditions at 1% O_2_ were necessary in the case of mouse (Fig. 8d). These results suggest that the TH-dependent cell cycle deceleration in OPCs under hypoxic condition is a general event occurring in rodents.

## Discussion

The current study provides evidences that, under hypoxic culture conditions at less than 1.5% O_2_ in the presence of PDGF and TH, actively proliferating perinatal OPCs progressively slow their proliferation and acquire adult OPC-like characteristics. The mechanisms for the TH-dependent cell cycle deceleration in OPCs involve Runx1. Furthermore, results collected in P14 rats showed that A2B5^+^ OPCs showing decreased proliferative signals express greater levels of Runx1 protein, suggesting pivotal roles of Runx1 in the development of adult OPCs *in vivo*.

Our study raised an important question as to whether the hypoxic conditions used in our culture are physiologically relevant^11^. The magnitude of hypoxia (equivalent 1% O_2_; pO_2_ = 7.2 mmHg at 37 °C) is known to occur in embryos—in the thymus, kidney medulla and bone marrow^49, 50^. The Km value of heme oxygenase-2, an O_2_-sensing enzyme in the CNS, is 15 mM (pO_2_ = 11 mmHg at 37 °C)^51^, which is close to our hypoxic culture (1.5% O_2_; pO_2_ = 13.8 mmHg at 37 °C). While the O_2_ concentrations in the CNS under normal conditions are estimated to be approximately 2–5% O_2_^21, 50^, and a simulation study of O_2_ diffusion suggested that O_2_ concentrations in a tissue decrease by 10-fold at a distance of several cell diameters from the nearest capillary^52^. These previous observations led us to hypothesize that a considerable number of OPCs reside in hypoxic niches *in vivo*. When we injected pimonidazole into living rats, as many as 30% of the OPCs in the optic nerves were positively labeled (Supplemmentary Fig. S9b). Considering that pimonidazole preferentially labels proteins in cells at O_2_ concentrations less than 14 mM (pO_2_ = 10 mmHg at 37 °C)^45^, the current results suggest that the pimonidazole-labeled OPCs actually reside under hypoxic conditions *in vivo* that are comparable to culture condition with less than 1.5% O_2_. It has been shown that a hypoxic environment is necessary to maintain the quiescence of adult OPCs *in vivo* ^32^. The current results suggest that such hypoxic environment is also essential for the development of adult OPCs by mechanisms involving Runx1.

Runx1 is a member of the Runx protein family^53^. Members of this family can directly bind to DNA via a conserved DNA-binding motif, known as the runt domain. In the peripheral nervous system, the defect of Runx1 activity causes impaired responses to noxious stimuli^54^. On the other hand, little is known about Runx1 loss of function in the CNS^55^. However, increasing Runx1 protein expression inhibits the proliferation and promotes the maturation of not only olfactory ensheathing cells, non-myelinating axon-wrapping glial cells in the olfactory nerve^56^, but also microglia, although they are myeloid lineage cells, in the postnatal forebrain^57^. To the best of our knowledge, there have been no previous reports about Runx1 expression in oligodendrocyte lineage cells. Importantly, Runx1 is the master gene of the development of hematopoietic stem cells (HSCs)^23^ and is required for the differentiation of HSCs from hemogenic endothelium in embryos^58^, as well as for the inhibiting the proliferation of myeloid progenitors in the bone marrow after birth^59^. In mouse and human HSCs, the overexpression of Runx1 protein variants, Runx1b (the variant expressed in the TH-stimulated hypoxic OPCs) or Runx1c, induces HSC quiescence *in vivo* ^60, 61^. Additionally, the Hif1α protein level in HSCs has been shown to play a critical role in regulating the quiescence and the differentiation of these cells^62, 63^. Thus, it is not unreasonable to suggest that as in OPCs, HIFs may induce HSCs quiescence via inducing Runx1 gene expression in hypoxic niches in the bone marrow.

**Figure 9:**
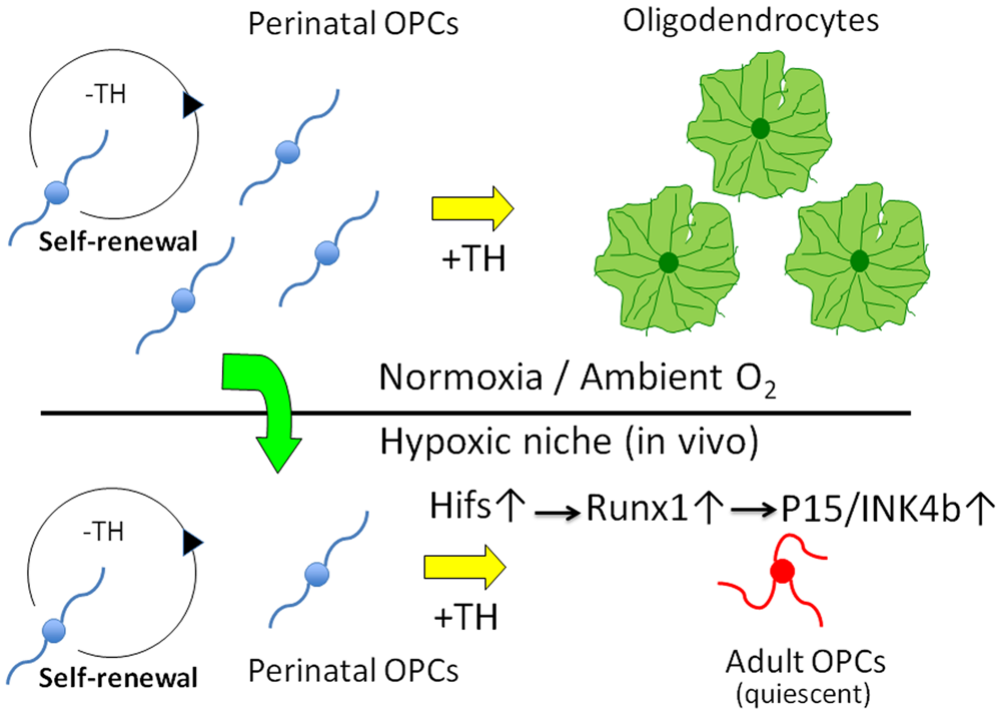
A schematic diagram of perinatal to adult transition of OPCs. In developing rat optic nerves, thyroid hormone inhibits the proliferation of perinatal OPCs. In normoxia, cells differentiate into oligodendrocytes. On the other hand, in a hypoxic niche, quiescent OPCs sustain their potential capacities of proliferation and differentiation as adult somatic stem cells (Adult OPCs). Hypoxia-inducible factors-mediated Runx1 expression and resultant p15/INK4b induction contributes to development of adult OPCs.

Our studies showed that p15/INK4b is the major effector molecule for the TH-dependent cell cycle deceleration in hypoxic OPCs, and its expression depends on the transcription factor Runx1, finally, the sensing mechanisms for environmental O_2_ concentrations by OPCs depend on the HIFs-mediated up-regulation of Runx1 gene expression. The current results shed light on the possible involvement of an unidentified, intrinsic cellular developmental program resembling the intracellular developmental timer that determines the timing of the OL differentiation of perinatal OPCs cultured in 20% O_2_^3^. Several lines of observation support this hypothesis. First, both the perinatal-to-adult transition of OPCs cultured in 1.5% O_2_ and the OL differentiation of OPCs in 20% O_2_ culture depend on extracellular PDGF and TH. Second, in the experiments using P7 rat optic nerve OPCs, the timing for the transition of OPCs from perinatal to adult-like in 1.5% O_2_ culture was almost identical to that for the differentiation into OL in 20% O_2_ culture (Fig. 1c). Third, in 20% O_2_ culture, TH can be replaced by retinoic acid (RA) to induce the time-dependent OL differentiation^16^. The cell cycle deceleration in OPCs in 1.5% O_2_ was also induced by adding RA (Supplementary Fig. S10). Revealing the mechanisms for the intrinsic cellular developmental program that determines the timing of the TH-dependent differentiation of adult-like OPCs under hypoxia deserve further investigation. Our findings are summarized in Fig. 9.

## Materials and Methods

All experiments in this study were carried out in accordance with the regulations of Keio University School of Medicine. The ethics committee of Keio University School of Medicine approved the current protocols for the animal experiments (ID; 09201-1). And the recombinant DNA experiments were carried out in accordance with the guidelines of the Ministry of Education, Culture, Sports, Science and Technology of Japan.

### Preparation of rodent OPCs

Sprague-Dawley (SD) rats and C57BL/6 mice were obtained from Japan SLC Inc. (Hamamatsu, Japan). The immunopanning purification of OPCs from rat optic nerves was performed according to Barres *et al*.^26^. In our experiments, over 97% of the cells were A2B5^+^ OPCs. The immunopanning purification of OPCs from rat cerebral cortex was performed according to Dugas *et al*.^47^. And the immunopanning purification of OPCs from mouse optic nerves was performed according to Watkins *et al*.^48^.

### Clonal density culture of OPCs

For clonal density cultures, 1,000–2,000 of purified OPCs were inoculated in poly D-lysine (PDL)-coated T25 culture flasks or slide flasks in serum-free modified BS medium^16^, containing 10 ng/ml PDGF, 5 ng/ml NT-3, 5 μg/ml insulin, 5 μM forskolin (20 μM for mouse OPCs)^64^, with or without TH (a mixture of 3,3′,5-triiodo-L-thyronine and thyroxine; 40 ng/ml of each). Cells were cultured at 37 °C in 5% CO_2_ and 1~20% O_2_ (in hypoxic culture conditions, O_2_ was replaced with N_2_).

### Immunocytochemistry

The procedures of immunocytochemistry were described previously^19^. Nucleuses were stained with DAPI (1 μg/ml) or PI (2 μg/ml) containing RNase A (50 μg/ml). Samples were examined with a BZ-9000 inverted fluorescence microscope (Keyence). Anti-GFAP rabbit polyclonal antibodies (G4546, Sigma-Aldrich), anti-MBP rat monoclonal antibody (MAB386, Millipore), anti-Ki-67 rabbit polyclonal antibodies (AB9260, Millipore), anti-Hif1α rabbit polyclonal antibodies (NB100-479, Novus Biologicals), anti-Hif2α rabbit polyclonal antibodies (NB100-122, Novus Biologicals), Anti-CDKN2B (p15/INK4b) rabbit polyclonal antibodies (bs-4269R, Bioss Antibody), anti-RUNX1/AML1 rabbit polyclonal antibodies (ab23980, Abcam).

### Viability assay

Nuclear staining dyes PI (2.5 μg/ml) and Hoechst 33342 (5 μg/ml) were added to the culture for 90 minutes. PI^+^/Hoechst 33342^+^ double-positive cells were considered dead.

### SA-βGal assay

SA-βGal assay was carried out obeying Debacq-Chainiaux *et al*.^30^. Cells were fixed with 2% PFA. They were rinsed and incubated in staining solution (40 mM citric acid/Na phosphate buffer (pH 6.0), 5 mM K_4_[Fe(CN)_6_], 5 mM K_3_[Fe(CN)_6_], 150 mM NaCl, 2 mM MgCl_2_, 1 mg/ml X-gal) for 16 hours at 37 °C. After then, cells were post-fixed with methanol. The samples were examined with an inverted microscope CKX41 (Olympus).

### BrdU incorporation assay

The procedures of the BrdU incorporation assay were described previously^19^. In short, cells cultured with 5 μM of BrdU were fixed and permeabilized in cold 70% ethanol, and incubated in 6N HCl-1% Triton-X100 for 15 minutes at room temperature (RT). After the rinse, cells were neutralized with 0.1 M sodium borate (in PBS with 1% Triton-X100) for 10 minutes at RT. Then cells were blocked with 50% normal goat serum-1% Triton-X100 and incubated with anti-BrdU rat monoclonal antibody (OBT0030, Oxford Biotechnology), followed by Alexa Fluor 488 goat anti-rat IgG antibodies. After post-fix, the nucleuses were stained with Hoechst 33342 (5 μg/ml).

### Movies

Time-lapse differential interference microscopic images of cells cultured on PDL/fibronectin (FN)-coated glass base culture dishes were recoded every 30 minutes for 5 days (120 hours) or every 15 minutes for 24 hours by using an incubator microscope LCV110 (Olympus). Editing of video images were carried out using Meta Imaging Software version 6.1 (Molecular Devices).

### Gene expression DNA Chip analysis

P7 rat optic nerves OPCs were cultured with or without TH in 1.5% O_2_ conditions for 15 days. Preparation of total RNA from OPCs was carried out using TRIZOL Reagent (Life Technologies) obeying manufacture’s instructions. The data analysis of Agilent Rat Gene Expression Microarray (Rat GE 4 × 44 k v3, 26,930 Entrez gene RNAs, Agilent Technologies) was carried out using GeneSpring13.1 software (Agilent Technologies).

### Reverse transcription-PCR (RT-PCR)

Total RNA purification was carried out using NucleoSpin RNA XS kit (Macherey-Nagel GmbH & Co.). SMARTer PCR cDNA synthesis kit (Clontech) was used for the cDNA synthesis obeying supplier’s instructions. For the preparation of PCR reaction mixture, PrimeStar GXL DNA polymerase kit (Takara-Bio) was used. The sequences of all PCR primers are shown in Supplementary Table S2. The condition of PCR was 10 seconds at 98 °C for the denaturing, 15 seconds at 60 °C for the annealing and 40 seconds at 68 °C for the extension. The total numbers of cycling were 20–35. The PCR products were analyzed on 2% agarose gel electrophoresis.

### Quantitative RT-PCR (qRT-PCR)

For the isolation of total RNA from OPCs, TRIZOL Reagent (Thermo Fisher Scientific) was used. Takara PrimeScript II 1st strand cDNA synthesis kit (Takara-Bio) was used for cDNA synthesis obeying supplier’s instructions. 20 μl of qRT-PCR reaction mixture consisted of 10 μl of BIO-RAD iQ SYBR Green Supermix (BIO-RAD), 5 μl of primer mix (2 μM of each forward and reverse primers) and 5 μl of template cDNA (0.2 ng/μl). The sequences of PCR primers for qRT-PCR are shown in Supplementary Table S3. Real-time PCR was performed on a CFX96 Real-Time System (BIO-RAD). The condition of PCR was 5 seconds at 95 °C for the denaturing, 30 seconds at 60 °C for the annealing and the extension. The total numbers of cycling were 40. The resulting values were normalized to the endogenous control gene, glyceraldehyde 3-phosphate dehydrogenase (*Gapdh*) or β-actin (*Actb*). Results were presented as the relative expression to that of the control using the comparative Ct method (∆∆Ct).

### siRNA mediated gene silencing

The sequences of the target sites of each siRNA are shown in Supplementary Table S4. The inhibitory efficiency on the protein expression by anti-Runx1, anti-Hif1α, anti-Hif2α, anti-p15/INK4b siRNAs is shown in Supplementary Fig. S4. Co-transfection of siRNA and green fluorescent protein (GFP) expressing reporter plasmid DNA pMaxGFP (Lonza) to OPCs was carried out using Amaxa Basic Glial Cells Nucleofector kit (Lonza) obeying supplier’s instructions. P7 rat OPCs cultured without TH in 1.5% O_2_ for 14 days were dissociated with trypsin. 3.3 × 10^5^ cells were suspended with 100 μl of Nucleofector solution mixture. Then, 2 μl of siRNA mixture (1 μM each) and 2 μl of GFP-expressing reporter plasmid pMaxGFP DNA (0.5 mg/ml) were added to it. Electroporation was operated using Nucleofector II (Lonza) programmed with #O-005. Cells suspended with 9 ml of TH-free BS medium were sifted using a 40 μm cell strainer. 3 ml of cell suspensions were inoculated into each PDL-coated slide flask. Cells were cultured in 1.5% O_2_ for 2 hours to allow them attaching the bottom, then all medium were replaced to 3 ml of fresh BS medium with TH. Cells were cultured in 1.5% O_2_ for 4–5 days. The number of cells in the GFP-expressing clones was counted.

### Construction of retrovirus vector plasmid DNA

Constructs of retrovirus vectors and the scheme of pINK4b-IRES-ZsGreen are shown in Supplementary Fig. S4b.

### Retrovirus vector infection

Preparation of retrovirus vectors was described previously^20^. Virus particles were added to the 2.0 × 10^4^ of freshly prepared P7 rat OPCs that had been cultured on PDL-coated T25 flasks with 5 ml of TH-free BS medium containing 10 μg/ml of protamine sulfate. Cells were cultured in 3% O_2_ for 4 hours to allow virus infection, then the cells were dissociated with trypsin and re-inoculated on PDL-coated slideflasks in clonal density and cultured for 5–7 days. Cells were fixed and stained with DAPI. The number of ZsGreen^+^/DAPI^+^ cells in each clone was counted.

### OPC sorting

P14 rat optic nerve derived anti-GC-panned cells^26^ were stained with AlexaFluor647-labeled A2B5 antibody (#563776, BD Biosciences). Stained cells were resuspended in staining medium with 1 μg/ml PI, and sorted by SORP FACSAria (BD Biosciences). Cells were attached to glass slides by Cytospin (Thermo Scientific) and were fixed with 4% PFA-PBS fixative. Each slide was stained with following primary and secondary antibodies: Alexa Fluor488-labeled anti-Ki67 mouse monoclonal antibody (1:100; #558616, BD Biosciences), anti-Runx1 rabbit polyclonal antibodies (1:100; ab23980, Abcam), and anti-rabbit IgG-Cy3 donkey polyclonal antibodies (1:500; 711-165-152, Jackson ImmunoResearch). Samples were incubated with primary antibodies overnight in humidified chambers at 4 °C. Secondary antibody and nuclear stain DAPI were placed on sections for 2 hours at RT. Samples were mounted with PermaFluor (Labvision), and analyzed by a laser-scanning confocal microscopy (FV-10; Olympus).

### Pimonidazole labeling *in vivo*

*In vivo* labeling of pimonidazole was carried out using Hypoxyprobe-1 kit (Hypoxyprobe, Inc.). P14 or P7 rats were administrated pimonodazole (60 mg/kg) via intraperitoneal injection^62^. Two hours later, animals were sacrificed and optic nerves were dissected within 5 minutes. 10,000 of optic nerve OPCs were suspended with 0.2 ml of TH-free BS medium and inoculated on PDL/gelatin-coated 12 mm glass base culture dishes and were cultured in 20% O_2_ for 90 minutes at 37 °C to allow them attaching the bottom. Cells were fixed with 4% PFA and were examined by immunocytochemistry.

### Statistics

In the case of the comparisons two, the data were evaluated statistically by Student’s *t* test. And one-way ANOVAs tests were used for multiple comparisons. Probability (P) < 0.05 was considered statistically significant. Error bars in each graph represent standard deviations (s.d.).

## Electronic supplementary material

Supplementary Information

Video 1

Video 2

Video 3

Video 4
