# Supplementary material for: Quiescence of adult oligodendrocyte precursor cells requires thyroid hormone and hypoxia to activate Runx1
Source: Sci Rep. 2017 Apr 21;7:1019. doi: 10.1038/s41598-017-01023-9 (PMC5430791; doi:10.1038/s41598-017-01023-9)
Supplement: Supplementary file 1 — Supplementary Information [file 41598_2017_1023_MOESM1_ESM.pdf]

Supplementary Information

Quiescence of adult oligodendrocyte precursor cells requires thyroid hormone and hypoxia to activate Runx1

Yasuhito Tokumoto[1]*, Shinpei Tamaki[1, 2], Yasuaki Kabe[1],
Keiyo Takubo[2], Makoto Suematsu[1¶]

1. Department of Biochemistry, Keio University School of Medicine, and Japan Science and Technology Agency (JST), Exploratory Research for Advanced Technology (ERATO), Suematsu Gas Biology Project, 35 Shinanomachi, Shinjyuku-ku, Tokyo 160-8582, Japan.
2. Department of Stem Cell Biology, Research Institute National Center for Global Health and Medicine, 1-21-1 Toyama, Shinjyuku-ku, Tokyo 162-8655, Japan

CONTACT
*Corresponding author:
Yasuhito Tokumoto, Ph. D.
TEL: +81-3-5363-3968
FAX: +81-3-5363-3466
e-mail: tokumoto@a2.keio.jp

¶Co-corresponding author:
Makoto Suematsu, M. D., Ph. D.
TEL: +81- 3-5363-3753
FAX: +81-3-5363-3466
e-mail: gasbiology@z6.keio.jp

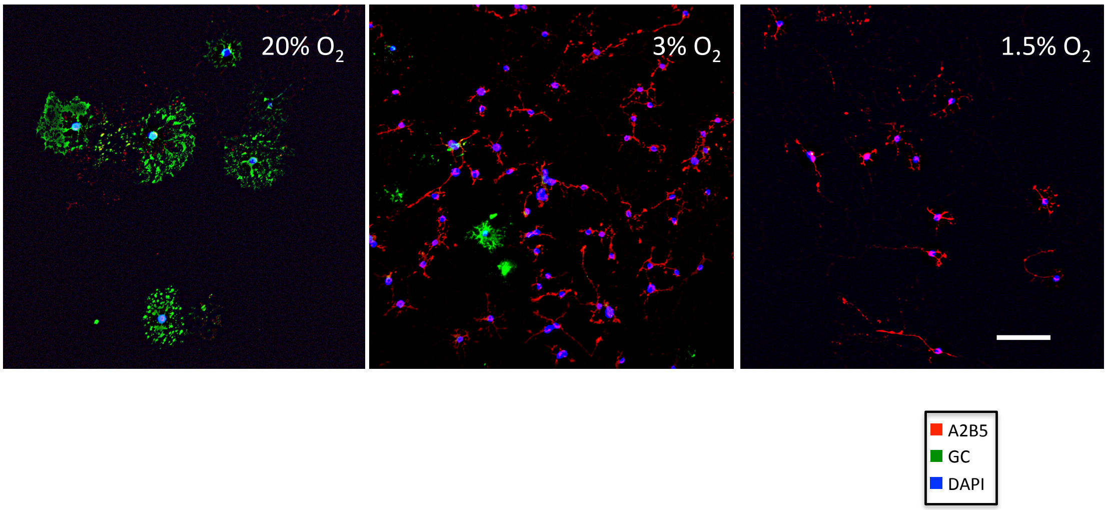

Supplementary Figure S1 Immunocytochemistry of P7 rat OPCs cultured with TH in 20%, 3% or 1.5%O_2.

Cells cultured with TH in 20%, 3% or 1.5% O_2 for 10 days were labeled with DAPI (*blue*), anti-GC antibody (*green*), and A2B5 antibody (*red*). Scale bar: 100 μm.

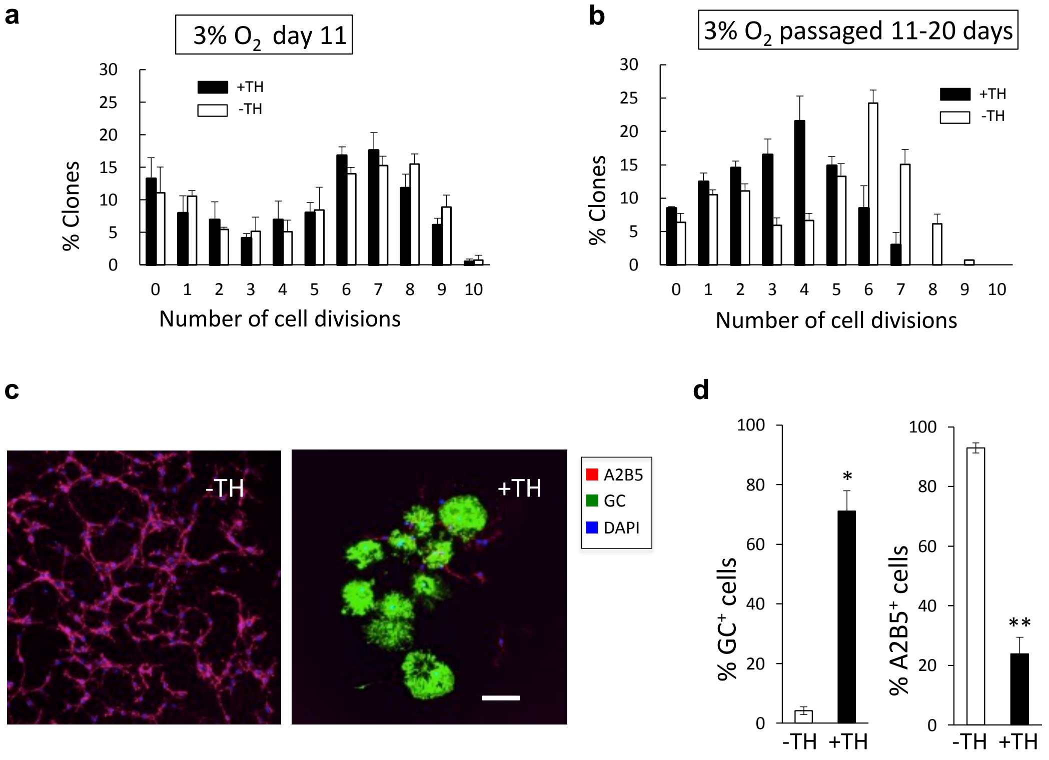

Supplementary Figure S2 The delayed timing of the TH-dependent OL differentiation in 3% O_2 culture.

(**a**) Clonal analysis of P7 OPCs cultured at clonal-density in PDGF in 3% O_2, with TH (*black bars*) or without TH (*white bars*). After 11 days, the number of cells in each clone was counted and the number of cell divisions was estimated (n = 3). (**b**) P7 OPCs were cultured with TH in 3% O_2 for 10 days and then removed and re-cultured at clonal-density in the same conditions for another 10 days; the clones were then assessed as in (a-c) (n = 3). (**c**) Cells in (b) were stained with the nuclear stain DAPI (*blue*) and a monoclonal anti-GC (*green*) or A2B5 (*red*) antibody (Scale bar: 100 μm), and (**d**) the % of the DAPI$^+$ cells that were GC$^+$ or A2B5$^+$ was determined. White bars; without TH (-TH), Black bars; with TH (+TH). P* < 0.01, P** < 0.01 (unpaired Student's *t*-test, n = 3). Note that most of the cells cultured in TH become GC$^+$ and A2B5$^-$ OLs, whereas most of those cultured without TH remained GC$^-$, A2B5$^+$ OPCs.

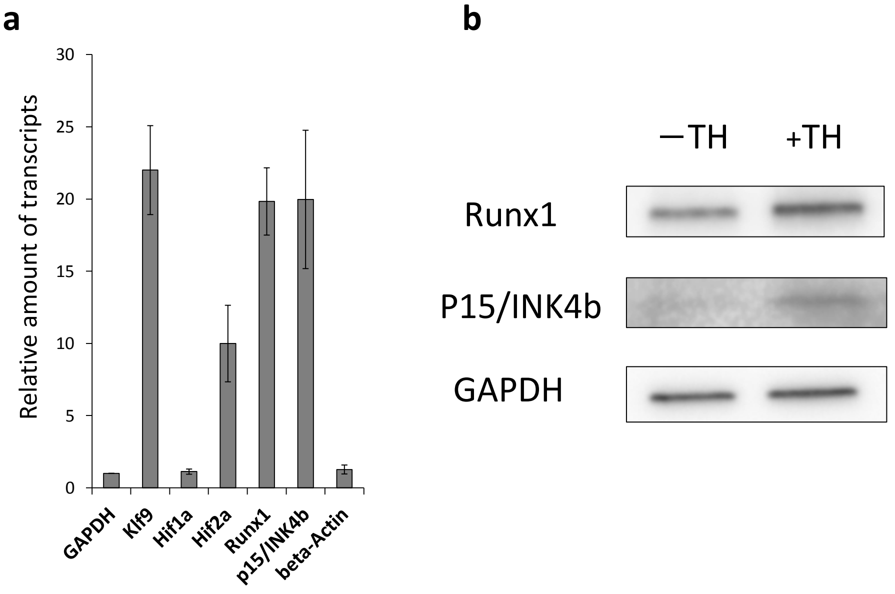

Supplementary Figure S3 Confirmation of TH-dependent gene up-regulations and TH-dependent protein accumulations in adult-like OPCs.

a. To confirm the TH-dependent gene up-regulation of Klf9, Hif2α, Runx1 and p15/INK4b, a qRT-PCR assay was carried out. Total RNA was prepared from P7 rat optic nerve OPCs cultured with or without TH in 1.5% O_2 for 15 days. The resulting values were normalized to the endogenous control gene GAPDH. Results of TH-stimulated cells are presented as the relative expression to those of the non-stimulated cells. Hif1α and beta-actin are also shown as negative controls. **b.** To confirm the TH-dependent accumulation of Runx1 protein and p15/INK4b protein in OPCs, P7 rat optic nerve OPCs were cultured with or without TH in 1.5% O_2 for 15 days, and a western blot analysis was carried out. The detecting primary antibodies were anti-Runx1 mouse monoclonal antibody (sc-365644, Santa Cruz) and anti-p15/INK4b (M-20) goat polyclonal antibodies (sc-1429, Santa Cruz). Same membrane was re-blotted with anti-GAPDH rabbit monoclonal antibody (#2118, Cell Signaling Technology) for internal control. -TH; non-stimulated OPCs, +TH; TH-stimulated OPCs. By the addition of TH, Runx1 protein increased to 1.4 folds and p15/INK4b protein increased to 9.8 folds. The methods: The imaging of the western blotting sheets was carried out by using an Image Quant LAS 4000(GE Health Care). The quantification of each band was carried out by using ImageQuant TL software (GE

Health Care) and with the sequential procedures as mentioned below. (1) Draw a rectangle box around the band of interest (Box A). (2) Copy the rectangle box and paste it on just above or below the area where without any bands in the correspond lane (Box B). (3) Measure the area intensities of both Box A and Box B. (4) To calculate the intensity of the band of interest, subtract the value of Box B (as the background) from that of Box A.

a

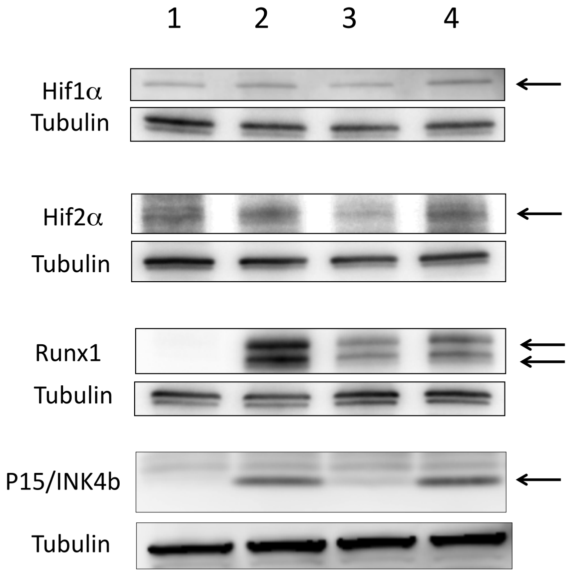

b

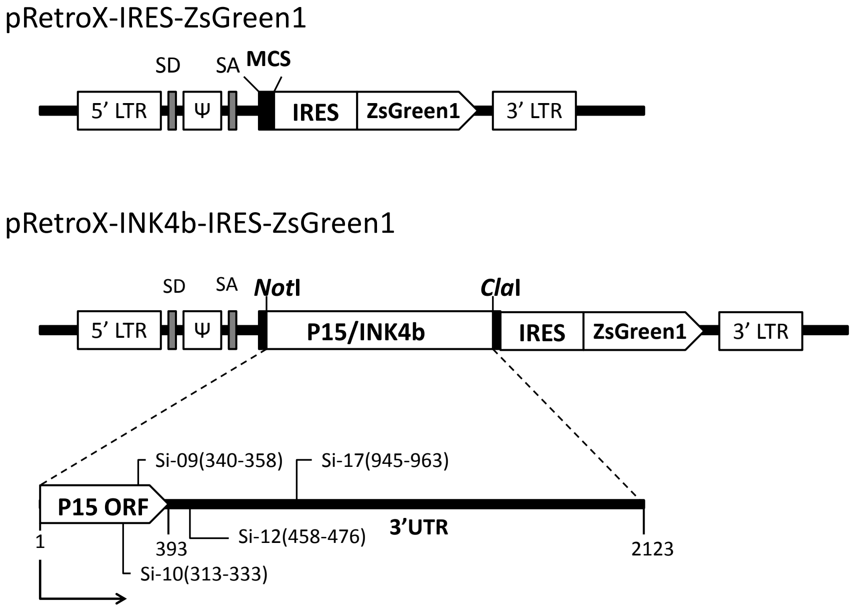

Supplementary Figure S4 The gene silencing efficiencies of anti-Runx1 siRNA, anti-Hif1α siRNA and anti-Hif2α siRNA, and anti-p15/INK4b siRNA,.

a. Human HEK293 cells were co-transfected with either 5 μg of target gene expressing

vector DNA (recombinant retrovirus vector plasmid DNA containing cDNA of each gene described in **b**) or 5 μg of empty vector DNA (pRetroX-IRES-ZsGreen1 retrovirus vector plasmid DNA, Clontech) and 200 pmol of siRNA against to each gene or non-target siRNA (shown in **Supplementary Table 4**). The cells were then cultured for 20 hours in 20% O_2. After harvesting, cell lysates were prepared and a series of western blotting analysis were carried out. The detecting primary antibodies were anti-Runx1 mouse monoclonal antibody (sc-365644, Santa Cruz), anti-p15/INK4b (M-20) goat polyclonal antibodies (sc-1429, Santa Cruz), anti-Hif1α rabbit polyclonal antibodies (NB100-479, Novus Biologicals) and anti-Hif2α rabbit polyclonal antibodies (NB100-122, Novus Biologicals). Same membrane was re-blotted with anti-tubulin rabbit polyclonal antibodies (3708-100, BioVision) for internal control. Lane 1; cells transfected with empty vector DNA, lane 2; with target gene expressing vector DNA, lane 3; with siRNA for each target genes and target gene expressing vector DNA, lane 4; with non-target siRNA and target gene expressing vector DNA. Black arrows show the positions of the band of the protein of target genes. Note; because of the cross reactivities of primary antibodies against the human homologues, the bands of Hif1α and Hif2α proteins appeared in the samples prepared from cells transfected with empty vector DNA (lane 1). The inhibitory efficiencies of protein expressions of each siRNA were; 52.6 ± 11.3% (n = 3) for anti-Hif1α siRNA, 53.7 ± 16.9% (n = 3) for anti-Hif2α siRNA, 55.8 ± 9.6% (n = 3) for anti-Runx1 siRNA, 68.6 ± 15.9% (n = 3) for anti-p15/INK4b siRNA. **b.** Structure of pINK4b-IRES-ZsGreen recombinant plasmid DNA and the efficiency of siRNA mediated gene silencing of p15/INK4b. Full length cDNA of rat p15/INK4b was cloned into NotI-ClaI site of the multiple cloning sites in pRetroX-IRES-ZsGreen1 vector plasmid DNA. The target sites of anti-p15/INK4b siRNA (**Supplmentary Table 4**) are shown. LTR: long terminal repeat, SD: splice donor site, SA: splice acceptor site, Ψ: packaging signal, MCS: multiple cloning site, IRES: encephalomyocarditis virus derived internal ribosome entry site, ORF: open reading frame.

For the Construction of retrovirus vector plasmid DNA, 1.5 kb of p15/INK4b cDNA fragment, 0.8 kb of Klf9 cDNA fragment and 1.4 kb of Runx1b isoform cDNA fragment were amplified by PCR from rat OPC cDNA library. The forward primer for p15/INK4b was 5'-GGGGCGGCCGCATGTTGGGCGGCGGCAG-3', and the reverse primer for p15/INK4b was 5'-TATATCGATTAAAAGGACAAGCAGTGAATAC-3'.

The forward primer for Runx1b isoform cDNA was 5'-GGGGCGGCCGCATGCGTATCCCCGTAGATGC-3', and the reverse primer for Runx1b isoform was 5'-CCATCGATTCAGTAGGGCCGCCAGACA-3'. The forward primer for Hif1α was 5'-GCCGCGGCCGCCACCGATTCGCCATG-3', and the reverse primer for Hif1α was 5'-CCATCGATGTACAGTTAGTGTTAGACCCAACTAC-3'. The forward primer for Hif2α was 5'-GTAGCGGCCGCACGGGGTTAAGGAACCC -3', and the reverse primer for Hif2α was 5'-CCATCGATCCCTGGCTCAGGTGG-3'. The forward primer for Klf9 was 5'-CCCGCGGCCGCACCATGTCCGCGGC-3', and the reverse primer for Klf9 was 5'-CCATCGATCTCACAAGGGGCTGGC-3'. The PCR reaction condition was same as RT-PCR. To construct pINK4b-IRES-ZsGreen plasmid DNA, the double strand PCR product was digested with restriction enzymes NotI and ClaI and then subcloned into the NotI-ClaI site in the multiple cloning sites of the pRetroX-IRES-ZsGreen1 retrovirus vector plasmid DNA. TOP10 chemically competent *E. coli* cells (Invitrogen) were transformed with recombinant plasmid DNA and cultured in LB medium containing 50 μg/ml of ampicillin at 37°C. Purification of plasmid DNA was carried out using an EndoFree Plasmid Maxi kit (QIAGEN) following the supplier's instructions. The nucleotide sequence was confirmed using a BigDye Terminator v1.1 cycle sequencing kit (Applied Biosystems).

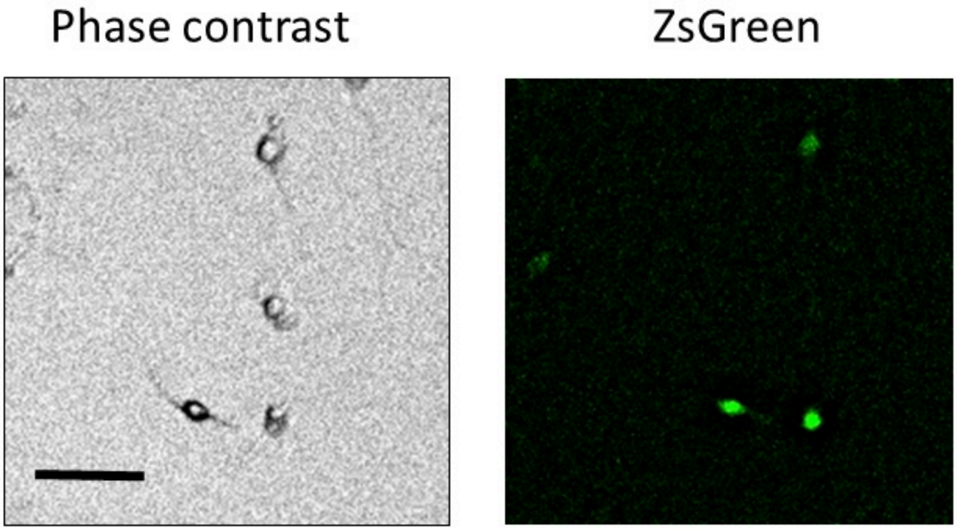

Supplementary Figure S5 Overexpression of Klf9 in P7 rat OPCs under hypoxia. Freshly prepared P7 rat OPCs were infected with a Klf9-IRES-ZsGreen expressing retrovirus vector were cultured at clonal-density in 1.5% O_2 and PDGF without TH for 7 days. The ZsGreen$^+$ cells are bipolar. Left panel; phase contrast, right panel; ZsGreen$^+$ (*green*). Scale bar: 50 μm.

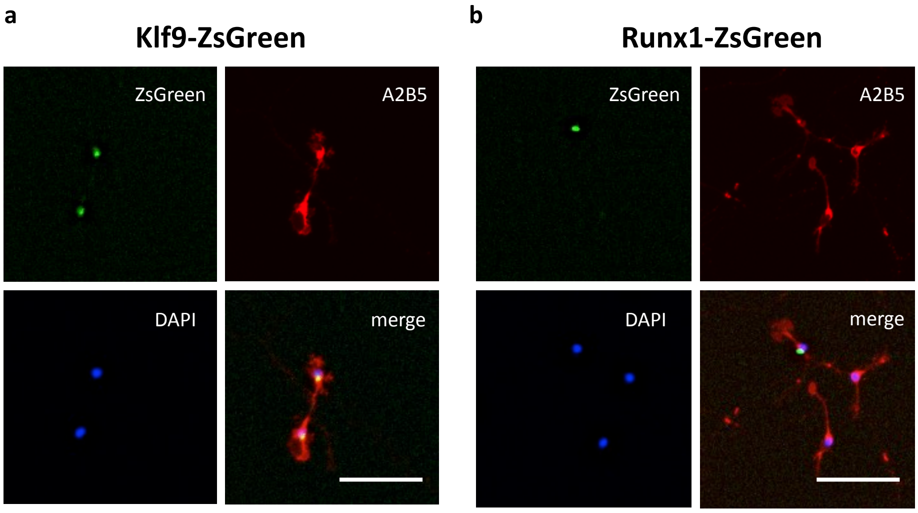

Supplementary Figure S6 Klf9 or Runx1 over expressing cells are A2B5^{+}.
P7 rat OPCs infected with retrovirus vector Klf9-IRES-ZsGreen (**a**) or Runx1-IRES-ZsGreen (**b**) were cultured without TH in 1.5% O_2 for 2 days and stained with A2B5 monoclonal antibody (*red*) and DAPI (*blue*) for nuclear staining. ZsGreen protein expressing retrovirus vector infected cells are shown in *green*. Scale bars: 100 μm.

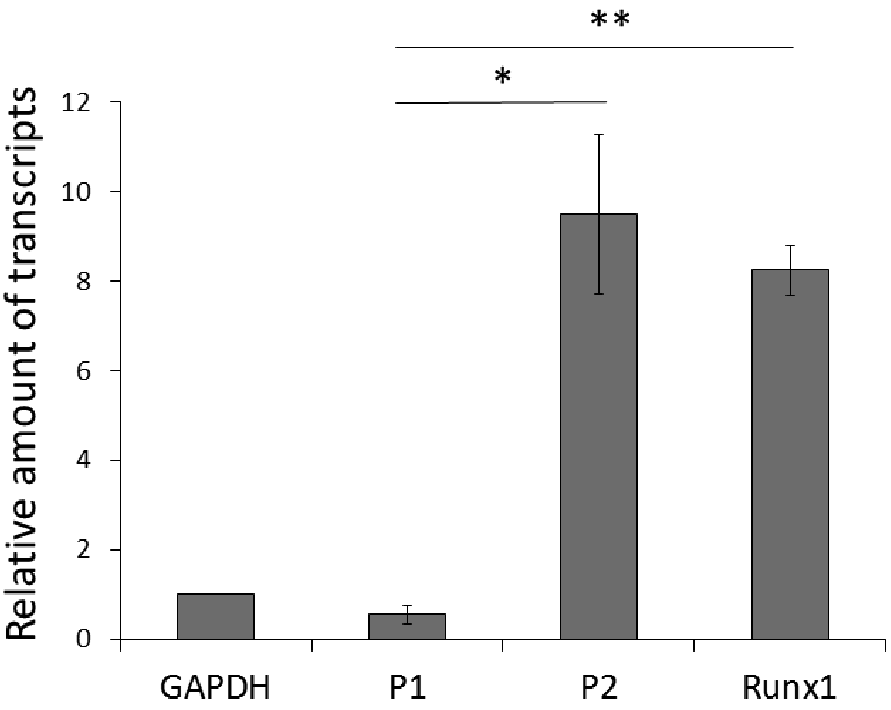

Supplementary Figure S7 TH activates the P2 promoter of Runx1 gene in hypoxic OPCs.

P7 rat OPCs were cultured in PDGF, with or without TH in 1.5% O_2 for 12 days. Total RNA was prepared from these cells and a qRT-PCR assay was carried out. The resulting values were normalized to the endogenous control gene *Gapdh*. Results of TH-stimulated cells are presented as the relative expression to those of the non-stimulated cells using the ΔΔCt methods. GAPDH; *Gapdh* specific PCR primer set, P1; *Runx1* transcripts derived from P1 promoter (Runx1c) specific PCR primer set, P2; *Runx1* transcripts derived from P2 promoter (Runx1b) specific primer set, Runx1; Common PCR primer set for Runx1c and Runx1b transcripts. P* <0.005, P** < 0.001 (ANOVA with Fisher's LSD test, n = 3).

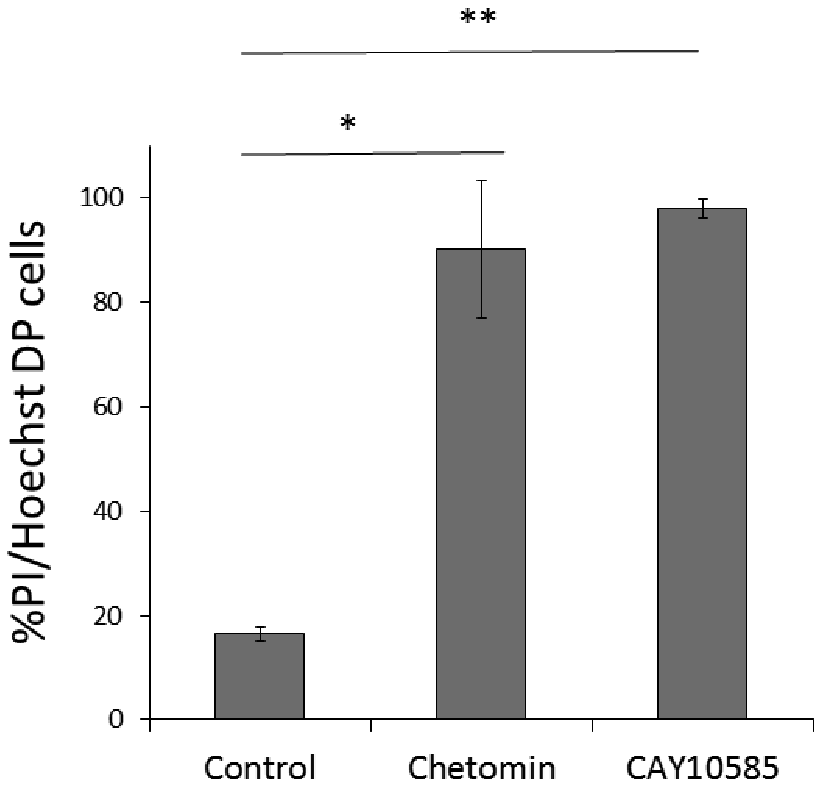

Supplementary Figure S8 Hifα inhibitors chetomin and CAY10585 induce cell death of OPCs under hypoxia.

P7 rat OPCs were cultured in PDGF without TH in 1.5% O_2 for 8 days. Then the medium was replaced to the TH containing medium. Cells were administrated with chetomin (2 μM) or CAY10585 (10 μM). For a control, 0.1% of DMSO was also added to the cultures. Cells were cultured for 24 hours and stained with nuclear staining dyes PI (2.5 μg/ml) and Hoechst 33342 (5 μg/ml). The number of cells was counted and PI^+/Hoechst 33342^+ double positive cells were considered as dead. In chetomin or CAY10585 containing culture, over 90% of OPCs died within 24 hours. P* <0.01, P** < 0.001 (ANOVA with Fisher's LSD test, n = 3).

a

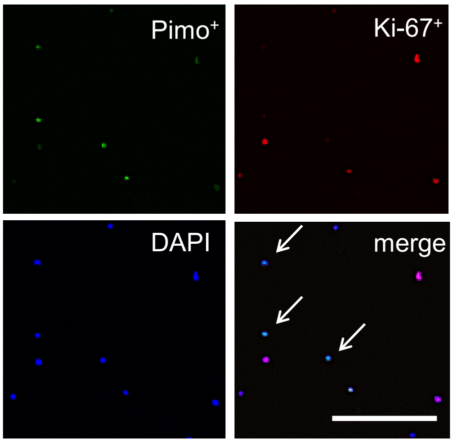

b

P14 rat OPC

Exp.	Pim$^+$/Ki-67$^+$	Pim$^+$/Ki-67$^-$	Pim$^-$/Ki-67$^+$	Pim$^-$/Ki-67$^-$	Total
1	109	128	299	215	751
2	116	165	376	305	962
3	94	116	252	181	643
4	134	156	360	263	913

P7 rat OPC

Exp.	Pim$^+$/Ki-67$^+$	Pim$^+$/Ki-67$^-$	Pim$^-$/Ki-67$^+$	Pim$^-$/Ki-67$^-$	Total
1	307	88	431	153	979
2	163	66	246	153	628
3	216	54	306	149	725

Supplementary Figure S9 ***In vivo*** **labeling of optic nerve OPCs with pimonidazole.**

(**a**) Pimonidazole was injected intraperitoneally into P7 rats or P14 rats. And after 2 hours, OPCs were purified from the optic nerves and labeled with a mouse monoclonal anti-pimonidazole antibody (*green*), rabbit anti-Ki-67 polyclonal antibodies (*red*) and DAPI (*blue*). Panels show the staining of P14 OPCs, white arrows show Pimo$^+$/Ki-67$^-$ cells; scale bar: 100 μm. (**b**) Cell count data for **Figure 7d**. Pim$^+$: anti-pimonidazole antibody positive, Ki-67$^+$: anti-Ki-67 antibody positive.

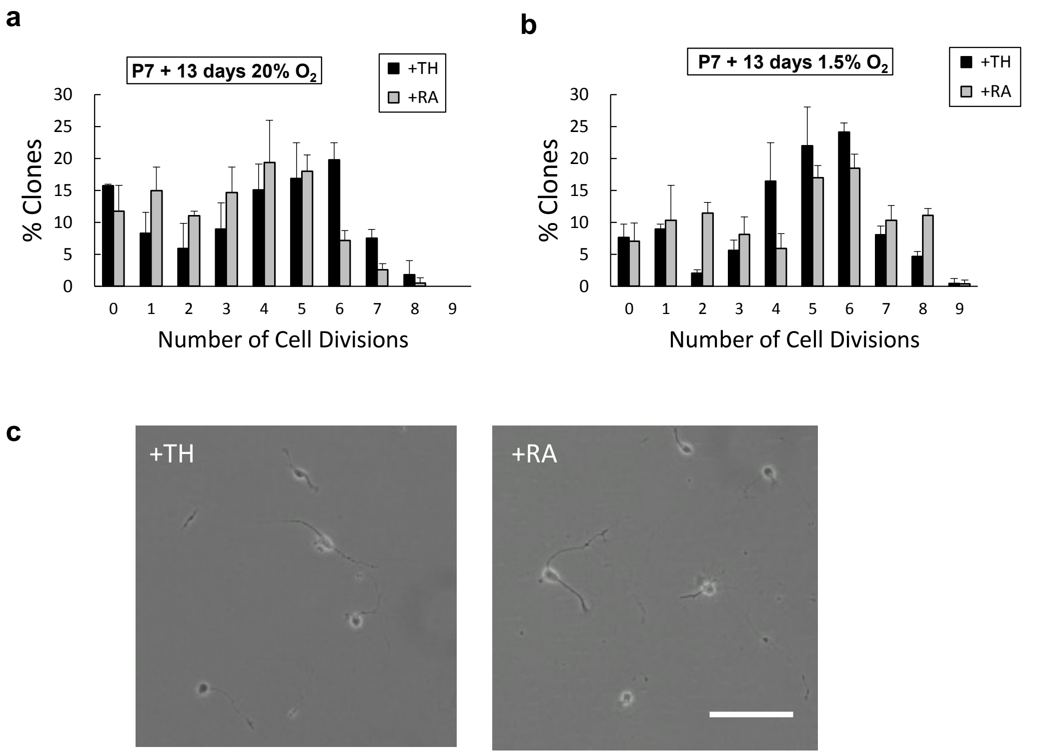

Supplementary Figure S10 The deceleration of cell cycle of OPCs in PDGF in 1.5% O_2 depends on in either TH or RA.

(**a and b**) P7 rat OPCs purified from optic nerves were cultured at clonal-density in PDGF, with either TH (*black bars*) or RA (1 ng/ml; *gray bars*) in either 20% O_2 (a) or 1.5% O_2 (b) for 13 days, and the number of cell divisions in each clone was estimated. (**c**) The phase-contrast images of P7 rat OPCs cultured for 13 days in 1.5% O_2 with TH (+TH; left panel) or RA (+RA; right panel) are shown. Scale bar: 100 μm.

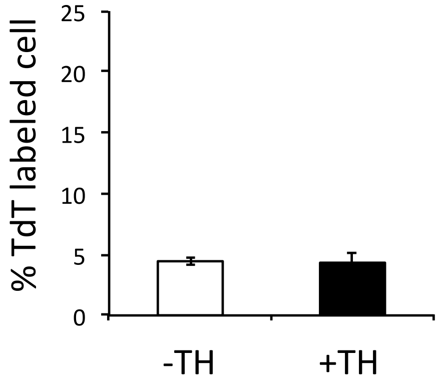

Supplementary Figure S11 Thyroid hormone did not increase the cell death of OPCs under hypoxia (a TUNEL assay).

2,000 of OPCs purified from P7 rat optic nerve were inoculated in each PDL-coated T25 flasks. These cells were pre-cultured in BS medium containing PDGF without TH for 14 days in 3% O_2 at 37℃. After then, OPCs dissociated and harvested. 10,000 of OPCs were re-inoculated on PDL/fibronectin coated 12 mm glass bottom dishes and cultured in BS medium containing PDGF without TH (*white bar*) or with TH (*black bar*) in 1.5% O_2 for 48 hours at 37℃. A TUNEL assay was carried out by using an In site Apoptosis Detection Kit (MK500; Takara Bio.) The percentage of terminal deoxynucleotidyl transferase (TdT)-labeled cells (dead cells) are shown (n = 3).

Genes Up-regulated (129)			
**Symbol**	**Raw data**	**Fold**	**Description**
Abca7	392	5.27	ATP-binding cassette sub-family A member 7
Adamts1	508	8.72	ADAM metallopeptidase with thrombospondin type 1 motif, 1
Apbb1ip	176	9.58	Rap1-GTP adapter molecule
Apln	117	23.92	Apelin, ligand for the G protein coupled receptor APJ
Apod	176	48.25	Apolipoprotein D
Arhgef28	773	5.12	Rho guanine nucleotide exchange factor 28
Bgn	220	13.96	Biglycan
Bmp4	3	119.81	Bone morphogenetic protein 4
Car2	26	37.53	Carbonic anhydrase 2
C1qtnf5	1,130	10.05	C1q and tumor necrosis factor related protein 5
Cacna1e	307	7.10	Voltage-dependent R-type calcium channel subunit alpha-1E
Caly	1,881	9.06	Calcyon, neuron-specific vesicular protein
Cav1	623	14.52	Caveolin 1
Cd9	202	12.20	CD9
Cdkn2b	1,177	7.72	Cyclin dependent kinase inhibitor p15/INK4b
Chn2	127	12.69	Chimerin 2
Chst5	218	12.12	Carbohydrate Sulfotransferase 5
Cldn11	54	28.49	Claudin 11
Clu	756	6.26	Clusterin (Apolipoprotein J)
Col9a2	612	5.44	Collagen α2 (IX)
Col11a2	65	29.74	Collagen α2 (XI)
Coro2b	121	42.08	Coronin, actin binding protein, 2B
Cryab	1,811	8.29	Crystallin, alpha B
Crym	185	436.66	Crystallin, mu, (NADP-regulated thyroid-hormone-binding protein)
Csrp1	222	17.92	Transcription factor, Csrp1

Ctsl1	1,627	8.34	Cathepsin L
Dbndd2	143	14.69	Dysbindin domain containing 2
Dbp	3,201	6.68	Transcription factor, Dbp
Dbx2	140	30.80	Transcription factor, Dbx2
Derl3	199	6.23	Derlin 3
Dynlt3	1,373	5.65	Dynein, light chain, Tctex-type 3
Epas1	132	14.82	Transcription factor, Hif2α
Espn	100	18.47	Espin, actin-bundling protein
Evi2a	731	6.27	Ecotropic viral integration site 2A
Fa2h	455	8.75	Fatty acid 2-hydroxylase
Fam134b	578	6.25	Family with sequence similarity 134, member B
Fbxo2	113	15.63	F-box protein 2
Fgfr2	130	44.39	FGF receptor 2
Galnt14	82	19.56	Polypeptide N-acetylgalactosaminyltransferase 14
Gfra2	237	21.52	GDNF family receptor alpha 2
Glul	1,622	8.55	Glutamate-ammonia ligase
Gpd1	70	91.66	Glycerol-3-phosphate dehydrogenase 1
Gpld1	119	9.44	Glycosylphosphatidylinositol specific phospholipase D1
Gpnmb	146	12.48	Glycoprotein (transmembrane) nmb
Gpr37	10	100.69	G protein-coupled receptor 37 (Pael-R)
Gpr37l1	8,348	6.93	G protein-coupled receptor 37 like 1
Gramd3	432	13.53	GRAM domain containing 3
Gsg1l	282	15.76	Germ cell associated 1-like
Gsn	2,037	5.09	Gelsolin
Hapln2	59	55.10	Hyaluronan and proteoglycan link protein 2
Hdac11	2,370	7.66	Histone deacetylase 11
Hist2h2aa3	2,002	5.17	Histone cluster 2, H2aa3
Hmox1	396	17.79	Heme oxygenase 1
Htra1	83	31.31	HtrA serine peptidase 1
Ifi27l2b	322	9.47	Interferon, alpha-inducible protein 27 like 2B

Igsf11	162	30.25	Immunoglobulin superfamily, member 11
Kcna1	917	7.84	Potassium channel, voltage gated shaker related subfamily A, member 1
Klf9	265	14.73	Transcription factor, Klf9
Lgals3	674	11.66	Lectin, galactoside-binding, soluble, 3
Lgi3	602	21.14	Leucine-rich repeat LGI family, member 3
Lhfp	2,017	9.72	Lipoma HMGIC fusion partner
Lhfpl2	341	19.89	Lipoma HMGIC fusion partner-like 2
Lppr5	356	6.19	Lipid phosphate phosphatase-related protein type 5
Mag	86	95.74	Myelin associated glycoprotein
Marc2	256	6.95	Mitochondrial amidoxime reducing component 2
Mbp	5,773	45.22	Myelin basic protein
Me1	7,802	8.89	Malic enzyme 1, NADP (+)-depended
Mettl7a	248	9.44	Methyltransferase like 7A
Mfsd6	1,487	5.48	Major facilitator superfamily domain containing 6
Mobp	18	192.83	Myelin-associated oligodendrocyte basic protein
Mog	10	163.83	Myelin oligodendrocyte glycoprotein
Myrf	91	6.61	Transcription factor, Mrf
Mro	173	10.14	Transcription factor, Mro
Myo1d	516	9.80	Myosin 1D
Ndrg1	16	59.35	N-myc downstream regulated 1
Neu4	894	5.13	Sialidase 4
Nfasc	419	8.75	Neurofascin
Ninj2	2,780	5.24	Ninjurin 2
Nkd1	280	8.20	Naked cuticle 1, Wnt antagonist
Nkx6-2	29	113.59	Transcription factor, Nkx6-2
Nr1d1	63	5.73	Transcription factor, Rev-erbα
Nrxn3	403	11.25	Neurexin 3
Oaf	134	14.25	Out at first homolog

Opalin	3	384.72	Oligodendrocytic myelin paranodal and inner loop protein
Pgf	88	102.47	Placental growth factor
Plcd4	203	12.09	Phospholipase C, delta 4
Plec	190	8.06	Plectin
Plekha1	3,504	5.43	Pleckstrin homology domain containing, family A member 1
Pllp	842	11.03	Plasmolipin
Plp1	221	146.97	Proteolipid protein 1
Ppp1r14a	22	58.95	Protein phosphatase 1, regulatory (inhibitor) subunit 14A
Ptchd2	191	8.67	Patched domain containing 2
Ptgds	18	256.55	Prostaglandin D2 synthase (brain)
Ptpre	248	7.20	Protein tyrosine phosphatase, receptor type, E
Rbp1	611	5.13	Retinol binding protein 1, cellular
Rgs7bp	973	9.38	Regulator of G-protein signaling 7 binding protein
Rpe65	3	244.49	Retinal pigment epithelium-specific protein 65kDa
Runx1	112	24.79	Transcription factor, Runx1
Sat1	1,033	7.96	Spermidine/spermine N1-acetyltransferase
Scg2	122	11.98	Secretogranin II, neuroendocrine secretory proteins
Scg5	372	6.23	Secretogranin V, neuroendocrine secretory proteins
Sds1	1,271	10.73	Solanesyl diphosphate synthase
Sema5a	50	22.96	Sema domain, seven thrombospondin repeats, transmembrane domain and short cytoplasmic domain, (semaphorin) 5A
Sept4	367	82.35	Septin 4, nucleotide binding protein
Serinc5	2,292	6.02	Serine incorporator 5
Shroom2	2,515	10.99	Shroom family member 2

Sirt2	1321	5.36	Sirtuin 2
Slc1a2	298	9.60	Excitatory amino-acid transporter 2
Slc6a3	488	11.81	Dopamine transporter
Slc7a3	7,801	5.35	Cationic amino acid transporter 3
Slc48a1	287	5.02	Heme transporter
Sparcl1	3,433	6.84	SPARC-like 1 (hevin)
Spsb1	698	8.26	SplA/Ryanodine receptor domain and SOCS box containing 1
Srpk3	504	6.32	SRSF protein kinase 3
Srxn1	312	6.43	Sulfiredoxin 1
Syt4	618	18.91	Synaptotagmin-4, Ca^{2+} sensor
Tf	72	29.44	Transferrin
Tmcc2	1,838	6.51	Transmembrane and coiled-coil domain family 2
Tmeff2	276	7.68	Transmembrane protein with EGF-like and two follistatin-like domains 2
Tmem100	179	9.86	Transmembrane protein 100
Tnr	260	80.08	Tenascin R
Tppp3	46	99.75	Tubulin polymerization-promoting protein family member 3
Tprn	582	5.65	Taperin
Tspan2	1,457	12.86	Tetraspanin-2
Tril	3,254	5.08	TLR4 interactor with leucine-rich repeat
Ttyh2	364	7.86	Tweety family member 2, calcium(2+)-activated large conductance chloride(-) channel
Tuba4a	79	21.27	Tubulin, alpha 4a
Ugt8	262	11.44	UDP glycosyltransferase 8
Vegfa	2,997	8.90	Vascular endothelial growth factor A
**Genes Down-regulated (60)**			
**Symbol**	**Raw data**	**Fold**	**Shown as**
Actg2	1,021	-8.54	Actin, gamma 2

Aif1l	1,271	-7.22	Allograft inflammatory factor 1-like
Arhgap11a	1,070	-6.01	Rho GTPase activating protein 11A
Aunip	554	-5.93	Aurora kinase A and ninein interacting protein
Aurkb	596	-6.41	Aurora kinase B
Brip1	616	-5.41	BRCA1-interacting protein 1
Bub1b	1,250	-5.19	BUB1 mitotic checkpoint serine/threonine kinase B
Cbx2	4,095	-5.20	Transcription factor, Cbx2
Ccna2	6,125	-6.08	Cyclin A2
Ccnb1	2,062	-6.25	Cyclin B1
Ccnb2	3,577	-5,21	Cyclin B2
Cdc6	845	-7.37	Cell division cycle 6
Cdc45	13,683	-6.21	Cell division cycle 45
Cdca2	861	-6.25	Cell division cycle associated protein 2
Cdca7	594	-14.51	Cell division cycle-associated protein 7
Cenpa	627	-6.94	Centromere protein A
Cenpm	1,260	-5.23	Centromere protein M
Cep55	1,748	-5.15	Centrosomal protein 55 kDa
Csrp2	2,110	-11.06	Transcription factor, Csrp2
Dll3	11,270	-14.34	Delta-like 3
Dsn1	1,044	-5.52	DSN1 homolog, MIS12 kinetochore complex component
Fam64a	7,236	-5.64	Family with sequence similarity 64, member A
Fgf13	565	-5.13	Fibroblast growth factor 13
Foxm1	604	-5.10	Transcription factor, Foxm1
Gins1	4,509	-6.34	GINS complex subunit 1 (Psf1 homolog)
Hist1h1b	11,437	-6.84	Histone cluster 1, H1b
Hmgb2	18,930	-5.77	High mobility group box 2
Igfbp4	6,386	-12.68	Insulin-like growth factor binding protein 4
Kif2C	1,211	-5.55	Kinesin family member 2C
Kif15	1,634	-5.17	Kinesin family member 15
Kif18a	571	-5.85	Kinesin family member 18A

Klf14	617	-5.20	Transcription factor, Klf14
Kntc1	1,345	-5.22	Kinetochore associated 1
Mcm3	11,644	-6.37	Minichromosome maintenance complex component 3
Mcm5	2,353	-6.55	Minichromosome maintenance complex component 5
Mcm6	3,608	-8.58	Minichromosome maintenance complex component 6
Melk	2,341	-5.27	Maternal embryonic leucine zipper kinase
Mis18a	2,879	-5.56	MIS18 kinetochore protein A
Mms22l	1,387	-6.33	MMS22-like, DNA repair protein
Mybl2	3,618	-6.00	Transcription factor, Mybl2
Ncaph	8,547	-5.06	Non-SMC condensin I complex, subunit H
Ns5atp9	2,547	-5.55	PCNA-associated factor (KIAA0101)
Prim1	4,030	-5.89	Primase, DNA, polypeptide 1
Racgap1	2,355	-5.42	Rac GTPase activating protein 1
Rad51	3,451	-5.28	RAD51 recombinase
Rasl11a	1,482	-5.53	RAS-like, family 11, member A
Rps6ka6	617	-5.47	Ribosomal protein S6 kinase, 90kDa, polypeptide 6
Rrm2	1,217	-7.71	Ribonucleotide reductase M2
Sdc1	6,488	-6.42	Syndecan 1
Sept6	666	-9.05	Septin 6, GTPase
Tac1	3,918	-5.27	Tachykinin, precursor 1, peptide hormone
Ticrr	744	-7.91	TOPBP1-interacting checkpoint and replication regulator
Timeless	1,843	-5.17	Timeless circadian clock
Tmpo	4,748	-5.28	Thymopoietin
Top2a	6,453	-5.81	Topoisomerase (DNA) II alpha
Tp53i11	693	-5.67	Tumor protein p53 inducible protein 11 (PIG11)
Tpx2	2,006	-5.28	TPX2, microtubule-associated

Ttk	4,389	-5.65	TTK protein kinase
Ung	810	-5.82	Uracil DNA glycosylase
Vim	38,812	-13.43	Vimentin
**Moderate genes described in the Text (11)**			
Ccnd1	21,272	+1.45	Cyclin D1
Ccnd2	4,783	+1.70	Cyclin D2
Ccnd3	1,145	+2.16	Cyclin D3
Ccne1	8,027	-0.40	Cyclin E1
Ccne2	250	-0.52	Cyclin E2
Cdkn1a	16,231	+2.38	Cip1/p21
Cdkn1b	4,289	-0.73	Kip1/p27
Cdkn1c	2,076	+1.08	Kip2/p57
Cdkn2a	4,311	+1.28	p16/INK4a & p19ARF
Cdkn2c	1,565	-0.39	P18/INK4c
Cdkn2d	2,269	-0.50	P19/INK4d

Supplementary Table 1 The list of differentially expressed genes with TH treatment.

Gene symbols are shown in *italic*. Average raw data of the P7 OPCs cultured without TH in 1.5% O_2 for 15 days are shown (n = 3). 129 Genes up-regulated with TH treatment are shown in (+) fold, and 60 genes down-regulated are shown in (-) hold. The moderate genes described in the text (11 genes) are also shown. For calculating probability (P value), Student's t test was carried out. The P value of every gene is $P < 0.05$ (*t*-test, n=3).

The TH-dependent up-regulations of the major genes described in the text were confirmed by qRT-PCR (**Supplementary Figure 3a**). And the TH-dependent up-regulations of protein expression of Runx1 and p15/INK4b were confirmed by western blot analysis (**Supplementary Figure 3b**).

Name of Gene	PCR Primer	Sequence (5'-3')	Length (mer)
p15/INK4b	FW	GCGGCAGTGACGCGGG	16
253 bp	REV	AGCGTGTCCAGGAAGCC	17
p16/INK4a	FW	TCACCAAACGCCCCGAAC	18
216 bp	REV	ATCGCGCACATCCAGCC	17
p19ARF	FW	TGGGTCGCAGGTTCGTGG	18
364 bp	REV	ATCGCGCACATCCAGCC	18
p18/INK4c	FW	GCAACTTACTAGTTTGTTGC	20
327 bp	REV	AGGCTGTGTGCTTCATTAG	19
p19/INK4d	FW	AGCTGGTGCATCCTGAC	17
310 bp	REV	GATGGAGATCAGATTCAG	18
p21/Cip1	FW	CAACTACGTCTGGGAGC	17
286 bp	REV	CTTGCAGAAGACCAATCG	18
p27/Kip1	FW	AGCTTGCCCGAGTTCTAC	18
308 bp	REV	TCCACAGTGCCAGCATTC	18
p57/Kip2	FW	CAGCCCACGGTTTTGTGG	18
291 bp	REV	AGAGGGATCCACGCATGG	18
Cyclin A2	FW	TTGCTGGAGCTGCCTTCC	18
278 bp	REV	ACAGCATGAACTCTGAGCG	19
Cyclin B1	FW	AACTCTGCAGCACTACCTG	19
247 bp	REV	AACTGCATCTGCAGACGAG	19
Cyclin B2	FW	AGAACAAATATGCCAGCAGC	20
297 bp	REV	TCTTCAGTACAGAGCACGG	19
Cyclin D1	FW	GGGAAGTTTTGTTGGGTCG	19
328 bp	REV	TCAGACATGGCCCTAAACC	19
Cyclin D2	FW	GCACAACGGCTCCAAGTC	18
310 bp	REV	CTGGACTTTGAACAGGTACC	20
Cyclin D3	FW	CAGGTAGAACCCACACACC	19
319 bp	REV	CCATCAGCACCCTCAAGAC	19
Cyclin E1	FW	TGTCAGAACAAAATAGGATTTC	22
318 bp	REV	TGGTGTTCTGGAGGTGGC	18

Cyclin E2	FW	ATGACACCACCGAAGAGCA	19
329 bp	REV	TTGGCTTCCATCCACTATTC	20
Csrp1	FW	GGGTAGAGCTGTGTTCTAG	19
270 bp	REV	TGATAAATCAGCTTCAACAGC	21
Dbp	FW	CTGAGGAGCCTTCTGCAG	18
616 bp	REV	TCAATTCCTCCTCTGAGAAG	20
Dbx2	FW	GGCAAGAGTTTCCTGATCG	19
538 bp	REV	TCTGGAACCAAATCTTCACC	20
Hif1α	FW	TGCTCATCAGTTGCCACTTC	20
309 bp	REV	TCCTCATGGTCACATGGATG	20
Hif1β	FW	GTCAGGGCTGGATTTTGATG	20
384 bp	REV	GCTGCCTCCAAAATCAAATG	20
Hif2α	FW	TGGACTACGAGGAGCCAG	18
405 bp	REV	TCACCTCACAGTCATATCTG	20
Klf9	FW	GAGGAGAGACAGGATTCTG	19
376 bp	REV	TGATCTCTTGATCATGCTGG	20
Mrf	FW	TACACTGGAGCCTGCTGC	18
334 bp	REV	ACTCTGCATGGACTCATGG	19
Mro	FW	TGTTCCTAAGGCGTCATCC	19
260 bp	REV	TGAGAAAGGAGGGATTGGG	19
Nkx6-2	FW	CTCAACGGGCTCGCCTC	17
356 bp	REV	CGCCGATTCTGGAACCAC	18
Rev-erbA	FW	TTCACGGCAGTGGTACTTG	19
368 bp	REV	GCCAGAGGCTCATCTTGG	18
Runx1	FW	AGCAACTCGCCCACCAAC	18
316 bp	REV	GCAATCAATAAGGTGCGGAA	20
GAPDH	FW	ACCACAGTCCATGCCATCAC	20
452 bp	REV	TCCACCACCCTGTTGCTGTA	20

Supplementary Table 2 The nucleotide sequences of RT-PCR primers.

The nucleotide sequences of PCR primers are shown from 5’ (left) to 3’ (right) direction. The numbers shown below each gene name are the length of predicted PCR products (bp). FW; forward primer, REV; reverse primer.

Primers for qRT-PCR			
GAPDH<br>108 bp	FW	TGGCATTGCTCTCAATGAC	19
	REV	TCCTTGGAGGCCATGTAG	18
Actb<br>63 bp	FW	TAAGGCCAACCGTGAAAAG	19
	RV	TACATGGCTGGGGTGTTG	18
Runx1<br>105 bp	FW	AGAAAGATTTTATACCCTTGACTTG	25
	RV	AAACAGGATGGGCTGC	16
Ldha<br>126 bp	FW	TCCAGTGTGTAAGTCCATCG	20
	REV	CATCATCCATATGCAGATCTTAC	23
Pgk1<br>115 bp	FW	AGCAGCTGCACAGCATC	17
	REV	AGATGCCCTCTACAATGCAC	20
Vegfa<br>106 bp	FW	GTGTGTGTGTATGAAATCTGTG	22
	REV	GGCAGAGCTGAGTGTTAGC	19
Klf9<br>108 bp	FW	CAACCAGGCAGAGATGG	17
	REV	AAAGAGCAGTGACCTCCTG	19
Hif1a<br>88 bp	FW	TGGACACTGGTGGCTC	16
	REV	CTAAACTATCTGAGTTGGTGCAG	23
Hif2a<br>109 bp	FW	GGATAACTTGTACCTGAAAGCC	22
	REV	CTGGGTAAGTCCCATGAAC	19
Runx1<br>P1 promoter<br>127 bp	FW	CACAGTGGTCAGCAGGCAG	19
	REV	AAGCCATCGTTTCCTTTCGA	20
Runx1<br>P2 promoter<br>120 bp	FW	CCTCCGGTAGTAATAAAGGCTCCTG	25
	REV	CCGATTGAGTAAGGACCCTGAA	22

Supplementary Table 3 The nucleotide sequences of qRT-PCR primers.
The nucleotide sequences of PCR primers are shown from 5’ (left) to 3’ (right) direction. The numbers shown below each gene name are the length of predicted PCR products (bp). FW; forward primer, REV; reverse primer.

Name of target gene	Name of siRNA	Target sequence
Non target control	Non-target 1	5’UGGUUUACAUGUCGACUAA3’
	Non-target 2	5’UGGUUUACAUGUUGUGUGA3’
	Non-target 3	5’UGGUUUACAUGUUUUCUGA3’
	Non-target 4	5’UGGUUUACAUGUUUUCCUA3’
p15/INK4b (*CDKN2B*)	J-093997-09	5’GAGCAGGGCCACCGUGAUA3’
	J-093997-10	5’CCGCCUGCCGGUAGACUUA3’
	J-093997-12	5’CUGUAAACGUGGAGAGGAU3’
	J-093997-17	5’GAUCUCUGCUAAACGGUGA3’
Csrp1 (*Csrp1*)	J-090904-08	5’UGAGAUCUAUUGCAAAGGA3’
	J-090904-07	5’CGACCACCCUGGCAGACAA3’
	J-090904-06	5’CAAGUCAUGUUAUGGCAAG3’
	J-090904-05	5’GCCGGGAAGUCCUGGCAUA3’
Dbp (*Dbp*)	J-089811-12	5’GCUAAUGACCUUUGAACCU3’
	J-089811-11	5’GGAAAGUCCAGGUGCCCGA3’
	J-089811-10	5’UGAAGGAAAAGGAGCGCAA3’
	J-089811-09	5’GUGCUGUGCUUUCACGCUA3’
Dbx2 (*Dbx2*)	J-108084-12	5’GAGCUGUGUUCUCGGAGGA3’
	J-108084-11	5’CUGUAUAAAUUCUCGUCUU3’
	J-108084-10	5’AGGGAAGAGUCUCCGGAAU3’
	J-108084-09	5’ACUAAGGAUCAAUGUGUAA3’
Hif1a (*Hif1a*)	J-091718-08	5’CUGAUAACGUGAACAAAUA3’
	J-091718-07	5’UUACUGAGUUGAUGGGUUA3’
	J-091718-06	5’GGAAACGAGUGAAAGGAUA3’
	J-091718-05	5’UGAGAGAAAUGCUUACACA3’
Hif2a (*Epas1*)	J-091647-12	5’AGGAUGAAGUGUACCGUCA3’
	J-091647-11	5’CCAUGGACACCGAGGCGAA3’
	J-091647-10	5’AGAUAGAGAAGAAUGACGU3’
	J-091647-09	5’UUGAUGAAUCCUCGACUUA3’
Klf9 (*Klf9*)	J-091801-08	5’GGAAAAUCCUCCCAUCUUA3’
	J-091801-07	5’GAAGGAUUAUUGCACACUG3’

	J-091801-06	5’GAACAAAUACCGACCCAUC3’
	J-091801-05	5’GGGAAACACGCCUCCGAAA3’
Mrf	J-085053-12	5’AAGAGGAAGCACUCCGAAU3’
(*Myrf*)	J-085053-11	5’GCAACAACAACAACGGCAU3’
	J-085053-09	5’UAGUGAACAAGGAGCGGAU3’
	J-085053-18	5’GAGCUGUGGUGGAGCGAAG3’
Mro	J-092502-11	5’GAGUUUCCAAAGUGAAGAA3’
(*Mro*)	J-092502-10	5’CUGCAUUUGCUGGGCGGAA3’
	J-092502-09	5’UGUCUUUAAUUUCGGAUGA3’
	J-092502-18	5’ACCCUGGAGUGCACGCAAA3’
Nkx6.2	J-085148-12	5’AGCACAAACCCUCGAACUU3’
(*Nkx6-2*)	J-085148-11	5’CGGAGGAUGAUGACGAGUA3’
	J-085148-10	5’AACUGGGACUCGCGGCAUA3’
	J-085148-09	5’CAGAGAUGGCGUCGGCUAA3’
Rev-erbA	J-097573-09	5’GCGUCAUUGUUCAACGUGA3’
(*Nr1d1*)	J-097573-10	5’GCCAGGGCCCAUCGAGAAA3’
	J-097573-11	5’GCUGAGAUGCAGAACGCCA3’
	J-097573-12	5’UAACCAAGCUUAACGGCAU3’
Runx1	J-092238-08	5’AGAACCAAGUGGCGAGAUU3’
(*Runx1*)	J-092238-07	5’GCGACGUGGUGGAAACCGA3’
	J-092238-06	5’GACCAUCACCGUCUUUACA3’
	J-092238-05	5’UGACCAGUCUUACCAGUAC3’

Supplementary Table 4 The sequences of siRNA target sites.

The symbol of gene is shown in (*Italic*). For the gene silencing experiments, a mixture of four siRNAs (1 μM each) specific to each target gene was used.

Legend of videos

Video 1. Perinatal OPCs without TH in 1.5% O_2 condition (as control).

OPCs purified from P7 rat optic nerves were cultured in the hypoxic condition without TH. These cells passaged once on day 14. On day 21, time-lapse differential interference microscopic images of cells were recoded every 15 minutes for 24 hours.

Video 2. Adult-like OPC with TH in 1.5% O_2 condition.

OPCs purified from P7 rat optic nerves were cultured in the hypoxic condition with TH. On day 21, time-lapse differential interference microscopic images of cells were recoded every 15 minutes for 24 hours.

Video 3. Adult-like OPCs differentiated into OLs in 1.5% O_2 condition.

300 of OPCs purified from P7 rat optic nerve were plated in BS medium containing PDGF with TH in 1.5% O_2 on PDL/fibronectin coated 12 mm glass-bottom dishes and pre-cultured at 37℃. On the culture day 10, the culture medium was replaced to PDGF free BS medium, after which (recording time 0) they were followed by time-lapse video microscopy. Images of cells were captured at every 15 minutes for 96 hours.

Video 4. Adult-like OPCs differentiated into type-2 astrocytes in 1.5% O_2 condition.

300 of OPCs purified from P7 rat optic nerve were plated in BS medium containing PDGF with TH in 1.5% O_2 on PDL/fibronectin coated 12 mm glass-bottom dishes and pre-cultured at 37℃. On the culture day 14, the culture medium was replaced to BS medium containing 10% FBS, after which (recording time 0) they were followed by time-lapse video microscopy. Images of cells were captured at every 20 minutes for 120 hours.

Supplementary Figure legends of Main Figures

Supplementary Figure Legend for Figure 1

(**a**) OPCs were purified from P7 rat optic nerve by immunopanning. 2,000 of OPCs were inoculated in each PDL-coated T25 flask. These cells were cultured in BS medium containing both PDGF and TH in 1.0~20% O_2 condition at 37℃ for 10 days. Then the cells were dissociated and harvested and the number of cells was counted. The P value of the cell number compared that of in 20% O_2 conditions are shown *P < 0.05 (ANOVA with Fisher's LSD test, n = 3), and compared that of in 2% O_2 conditions are shown $^{\vee}$P < 0.05 (ANOVA, n = 3).

(**b**) 1,000 of OPCs purified from P7 rat optic nerve were inoculated in each PDL-coated slide flask. These cells were cultured in BS medium containing both PDGF and TH in 1.0~20% O_2 condition at 37℃ for 10 days. After then, the cells were fixed and stained with anti-GC or A2B5 monoclonal antibody, and the percentage of GC^+ cells (*white bars*) and $A2B5^+$ cells (*black bars*) was determined (upper panel). The ratios of $A2B5^+$ versus GC^+ are shown in the lower panel. *P < 0.05 (ANOVA with Fisher's LSD test, n = 3).

(**c**) 2,000 of OPCs purified from P7 rat optic nerve were inoculated in each PDL-coated T25 flask. These cells were cultured in BS medium containing PDGF with TH (*black bars*) or without TH (*white bars*) in 1.5 or 20% O_2 condition at 37℃ for 11 days. The number of cells in each clone was counted and the number of cell divisions was estimated (n = 3).

(**d**) 1,000 of OPCs purified from P7 rat optic nerve were inoculated in each PDL-coated T25 flask. These cells were cultured in BS medium containing PDGF without TH in either 20% O_2 (*closed squares*) or 1.5% O_2 (*open squares*) at 37℃ and were passaged at day 10, day 20, day 30 and at day 40 (1,000 cells per T25 flask). Cell numbers estimated from the total cell number at the last passage multiplied by the proliferation rate (n = 3).

Supplementary Figure Legend for Figure 2

(**a**) 2,000 of OPCs purified from P7 rat optic nerve were inoculated in each PDL-coated T25 flask. These cells were cultured in BS medium containing both PDGF and TH in

either 20% O_2 (*closed squares*) or 1.5% O_2 (*open squares*) condition at 37℃. These cells were stained for GC after 3, 6 and 12 days, and the % of GC^+ cells were counted at each time point. *P < 0.05 (unpaired Student's *t*-test, n = 3).

(**b**) 1,000 of OPCs purified from P7 rat optic nerve were inoculated in each PDL-coated slide flask. These cells were were cultured in BS medium containing PDGF, without TH (-TH, *white bar*) or with TH (+TH, *black bar*), in 1.5% O_2 at 37℃. At culture day 15, PI (2.5 μg/ml) and Hoechst 33342 (5 μg/ml) were added to the culture for 90 minutes. Then, the percentage of dead cells (PI^+, Hoechst 33342^+ double positive cells) was determined (n = 3).

(**c**) 1,000 of OPCs purified from P7 rat optic nerve were inoculated in each PDL/fibronectin-coated 12 mm glass bottom dish. Cells were cultured in both PDGF and TH in 1.5% O_2 at 37℃ for 20 days and stained for SA-βGal (left panel); as a positive control, 1,000 of OPCs purified from P7 rat optic nerve were cultured in 15% FBS in 20% O_2 for 20 days to induce replicative senescence were also stained for SA-βGal (right panel). Scale bar: 50 μm.

(**d**) 10,000 of OPCs purified from P7 rat optic nerve were inoculated in each PDL-coated slide flask and cultured without PDGF at 37℃ for 3 days, in either 20% O_2 (upper right panel) or 1.5% O_2 (lower right panel). After then, cells were labeled with anti-GC antibody (*green*) and DAPI (*blue*). Scale bar: 100 μm. Percentages of GC^+ cells in 20% O_2 culture and 1.5% O_2 culture are shown in the graph on the left (n = 3). Note that rat OL differentiation obeying withdrawal of PDGF is not inhibited in 1.5% O_2, the extensions of GC^+ membrane-like structure around the cell body in 1.5% O_2 were comparable with those in 20% O_2.

Supplementary Figure Legend for Figure 3

(**a**) 1,000 of OPCs purified from P7 rat optic nerve were inoculated in each PDL-coated slide flask and cultured in BS medium containing PDGF with TH (+TH; black bars) or without TH (-TH; white bars) in 1.5% O_2 at 37℃. After 2 or 15 days, cells were treated with 5 μM of BrdU for 20 hours and then the % of $BrdU^+$ cells was determined. *P < 0.001 (unpaired Student's t-test, n = 3).

(**b**) 300 of OPCs purified from P7 rat optic nerve were plated in BS medium containing PDGF, with TH (*closed circles*) or without TH (*open squares*) and in either 1.5% or 3% O_2 on PDL/fibronectin coated 12 mm glass-bottom dishes and pre-cultured for 24 hours

at 37℃, after which (recording time 0) they were followed by time-lapse video microscopy. Images of cells were captured at every 30 minutes for 120 hours. The average time between M-phases was estimated on day 1 (recording time 0-23.5 hours), day 2 (24-47.5 hours), and day 3 (48-71.5 hours). At the recording time 0, the following number of cells were analyzed in each condition: 42 in 1.5% O_2 with TH; 48 in 1.5% O_2 without TH; 131 in 3% O_2 with TH; 89 in 3% O_2 without TH. *$P < 0.05$ (unpaired Student's *t*-test).

(**c**) 1,000 of OPCs purified from P7 rat optic nerve were inoculated in each PDL-coated slide flask and cultured in BS medium containing PDGF and TH in 1.5% O_2 at 37℃. At culture day 11, 20 or 30, the cells were fixed and labeled with anti-GC and A2B5 monoclonal antibodies and the percentage of each type of labeled cells was determined (n = 3).

(**d**) 500 of OPCs purified from P7 rat optic nerve were inoculated in each PDL-coated slide flask and cultured in BS medium containing PDGF and TH in 1.5% O_2 at 37℃. After 15 or 30 days, the representative fields were examined by phase-contrast microscopy. Scale bar: 50 μm.

(**e**) 500 of OPCs purified from P7 rat optic nerve were inoculated in each PDL-coated slide flask and cultured in BS medium containing PDGF and TH in 1.5% O_2 at 37℃ for 15 days. Before the differentiation inducing treatments, colonies in these flasks were examined by a phase-contrast microscopy. The positions of the colonies of which contained only OPCs with characteristic bipolar bodies were marked and labeled on the bottom of the slide flasks. Cultures were then either deprived of PDGF for 5 days and labeled for MBP or treated with 10% FBS for 5 days and labeled for GFAP. The morphologies of the cells and the expressions of MBP or GFAP in the cells of the marked colonies were examined. Scale bar: 100 μm.

(**f**) 2,000 of OPCs purified from P7 rat optic nerve were inoculated in PDL-coated T25 flasks. These cells were cultured in BS medium containing both PDGF and TH in 1.5% O_2 for 15 days at 37℃. Cells were dissociated with trypsin and re-cultured at clonal-density (2,000 cells/PDL-coated T25 flask) for another 7 days at 37℃ in PDGF in 20% O_2 — without TH (*white bars*), with TH (*black bars*), or without TH in the presence of NRG1 (50 ng/ml) and IBMX (100 μM) (*gray bars*). Then cells were fixed and the number of cell divisions in each clone was estimated. The average numbers of cell divisions are show in the inset (1.24 ± 0.18 without TH; 0.24 ± 0.60 with TH; 1.95

± 0.60 without TH, but with NRG1 and IMBX). *$P < 0.001$ (unpaired Student's *t*-test, n = 3).

Supplementary Figure Legend for Figure 4

(**a and b**) 2,000 of OPCs purified from P7 rat optic nerve were inoculated in each PDL-coated T25 flask. These cells were were cultured in BS medium containing PDGF, without TH (-TH) or with TH (+TH), in 1.5% O_2 at 37℃. At culture day 15, total RNA was prepared from these cells and and assayed by RT-PCR. The PCR products were detected by 2% agarose gel electrophoresis. GAPDH was used as an internal control. (a) In Cyclin genes, TH decreased the mRNA for G2/M Cyclins A2, B1 and B2. (b) In CKI genes, TH strongly increased the mRNA for p15/INK4b.

(c) 1,000 of OPCs purified from P7 rat optic nerve were inoculated in PDL-coated slide flasks. Cells were pre-cultured in BS medium containing PDGF without TH in 1.5% O_2 for 10 days at 37℃. Then TH (40 ng/ml) was added to some cultures (+TH) but not to others (-TH) for 2 days. The cells were then labeled with rabbit anti-p15/INK4b antibodies (*green*) and DAPI (*blue*). Note that p15/INK4b protein increased in the nucleus of the TH-treated cells. Scale bar: 100 μm.

(**d**) 2,000 of OPCs purified from P7 rat optic nerve were inoculated in PDL-coated T25 flasks. OPCs were pre-cultured in BS medium containing PDGF without TH in 3% O_2 for 12 days at 37℃. Cells were dissociated and harvested. These cells were co-transfected with anti-p15/INK4b siRNA (si-p15) and GFP expressing transfection-reporter plasmid DNA (pMaxGFP); a non-targeting siRNA pool (si-NT) served as a negative control. Cells were re-cultured in PDL-coated slide flasks at clonal-density with BS medium containing PDGF with TH in 1.5% O_2 for 5 days at 37℃. The number of GFP^+ cells in each clone was counted. Note that the anti-p15/INK4b siRNA prevented the deceleration of cell cycle that occurs in 1.5% O_2 in the presence of TH. *$P < 0.001$ (unpaired Student's *t*-test, n = 3).

(e) 20,000 of OPCs purified from P7 rat optic nerve were inoculated in PDL-coated T25 flasks. Cells were pre-cultured in BS medium containing PDGF without TH in 3% O_2 for 12 hours at 37℃. Then cells were infected with either a p15/INK4b-IRES-ZsGreen expressing retrovirus vector (p15) or the ZsGreen expressing empty vector as a negative control (Cont) for 4 hours at 37℃. Cells were dissociated with trypsin and re-inoculated at clonal density in PDL-coated slide flasks (500 cells/slide flask). Cells were cultured

in BS medium containing PDGF with TH in 3% O_2 for 7 days at 37℃. The number of ZsGreen$^+$ cells in each clone was counted, and the results are shown in the graph on left. Note that the cells over expressing p15/INK4b proliferated much less than control. *P < 0.001 (unpaired Student's *t*-test, n = 3). The p15INK4b-IRES-zsGreen expressing cells were also labeled with A2B5 antibody (*red*) and DAPI (*blue*); as shown in the panels on right, the ZsGreen$^+$ OPCs (*green*) are bipolar and A2B5$^+$. Scale bar: 100 μm.

Supplementary Figure Legend for Figure 5

(**a**) 2,000 of OPCs purified from P7 rat optic nerve were inoculated in PDL-coated T25 flasks. Cells were pre-cultured in BS medium containing PDGF without TH in 1.5% O_2 for 12 days at 37℃. Cells were dissociated and harvested. These cells were co-transfected with anti-p15/INK4b siRNA (si-p15/INK4b) or siRNA against the gene of each transcription factor and pMaxGFP. Cells were re-cultured in PDL-coated slide flasks at clonal-density with BS medium containing PDGF with TH in 1.5% O_2 for 4 days at 37℃. The number of GFP$^+$ cells in each clone was counted. Data was normalized against the average number of negative control (cells transfected with non-target siRNA; si-NT). P* <0.05, P** < 0.01, P***< 0.001 (unpaired Student's *t*-test, n = 3).

(**b**) 2,000 of OPCs purified from P7 rat optic nerve were inoculated in PDL-coated T25 flasks. Cells were pre-cultured in BS medium containing PDGF without TH in 1.5% O_2 for 10 days at 37℃. To investigate the immediate early responses of the genes of the deceleration of cell cycle related transcription factors, these cells were pre-treated with cycloheximide (50 μg/ml) for 6 hours, after then TH was added to the medium. Cells were harvested at 0, 0.5, 1 and 2 hours later. Total RNA was prepared from these cells and a RT-PCR assay was carried out. *Gapdh* (GAPDH) was used as a negative control. The PCR products were detected by 2% agarose gel electrophoresis.

(c) 20,000 of OPCs purified from P7 rat optic nerve were inoculated in PDL-coated T25 flasks. Cells were pre-cultured in BS medium containing PDGF without TH in 3% O_2 for 12 hours at 37℃. Then cells were infected with either Klf9-IRES-ZsGreen expressing retrovirus vector or the ZsGreen expressing empty vector as a negative control for 4 hours in 3% O_2 at 37℃. Cells were dissociated with trypsin and re-inoculated at clonal density in PDL-coated slide flasks (500 cells/slide flask). Cells were cultured in BS medium containing PDGF without TH (-TH) in 1.5% or 3% O_2

conditions for 7 days at 37℃. The number of ZsGreen$^+$ cells in each clone was counted, and the results are shown in the graphs. Cont; empty vector RetroX-IRES-ZsGreen 1 infected cells, Klf9; Klf9 over expressing cells. P* < 0.001 (unpaired Student's *t*-test, n = 3).

(d) 2,000 of OPCs purified from P7 rat optic nerve were inoculated in PDL-coated T25 flasks. Cells were cultured in BS medium containing PDGF without TH (-TH) or with TH (+TH) in 1.5% O_2 for 1, 4 and 15 days at 37℃, or in 3% O_2 for 15 days at 37℃. Total RNA was prepared from those cells and a RT-PCR assay was carried out. *Gapdh* (GAPDH) was used as a negative control. The PCR products were detected by 2% agarose gel electrophoresis.

(e) 20,000 of OPCs purified from P7 rat optic nerve were inoculated in PDL-coated T25 flasks. Cells were pre-cultured in BS medium containing PDGF without TH in 3% O_2 for 12 hours at 37℃. Then cells were infected with Runx1 (Runx1b-IRES-ZsGreen) over-expressing retrovirus vector (Runx1) or ZsGreen expressing empty vector (Control) for 4 hours in 3% O_2 at 37℃. Cells were dissociated with trypsin and re-inoculated at clonal density in PDL-coated slide flasks (500 cells/slide flask). These cells were cultured in BS medium containing PDGF with TH (+TH) or without TH (-TH) in 3% O_2 conditions for 7 days at 37℃. The number of ZsGreen$^+$ cells in each clone was counted, and the results are shown in the graphs. Control; empty vector RetroX-IRES-ZsGreen 1 infected cells, Runx1; Runx1 over expressing cells. The number of ZsGreen$^+$ cells in each clone was counted. P* <0.005, P** < 0.001 (unpaired Student's *t*-test, n = 3).

(f) OPCs overexpressing Runx1 (*green*) in (d) were stained with the nuclear stain DAPI (*blue*) and rabbit anti-p15/INK4b antibodies (*red*). Scale bar: 100 μm. By using the BZ-Ⅱ Analyzer software (Keyence), the fluorescent intensities of the staining of anti-p15/INK4b antibodies in each cells were estimated. The average staining intensity of Runx1-overexpressing cells was 22,743 ± 11,425 pxl (n = 29), that was 11-folds higher than that of the non-transformed cells (2,032 ± 3,862 pxl; n = 49).

Supplementary Figure Legend for Figure 6

(a) 2,000 of OPCs purified from P7 rat optic nerve were inoculated in PDL-coated T25 flasks. Cells were cultured in BS medium containing PDGF without TH (-TH) or with

TH (+TH) in 1.5% O_2 for 15 days at 37℃. Total RNA was prepared from these cells and a RT-PCR assay for the gene expressions of *Hif1a* (Hif1α), *Epas1* (Hif2α) and *Arnt* (Hif1β) was carried out. *Gapdh* (GAPDH) was used as a negative control. The PCR products were detected by 2% agarose gel electrophoresis.

(b) 1,000 of OPCs purified from P7 rat optic nerve were inoculated on PDL/fibronectin coated 12 mm glass-bottom dishes. Cells were cultured in BS medium containing PDGF without TH in 20% O_2 for 7 days, and some were then changed to 1.5% O_2 for 20 hours. The cells were labeled with rabbit anti-Hif1α antibodies (*green*) and PI (*red*). Scale bar: 50 μm.

(**c**) 1,000 of OPCs purified from P7 rat optic nerve were inoculated on PDL/fibronectin coated 12 mm glass-bottom dishes. Cells were cultured in BS medium containing PDGF, without TH (-TH) or with TH (+TH) in 1.5% O_2 for 10 days. The cells were labeled with rabbit anti-Hif2α antibodies (*green*) and PI (*red*). To prevent the degradation of Hif2α protein, some cultures were treated with 0.2 mM of $CoCl_2$ (Yuan *et al*., 2003) for last 7 hours. Scale bar: 50 μm.

(**d**) 2,000 of OPCs purified from P7 rat optic nerve were inoculated in PDL-coated T25 flasks. Cells were pre-cultured in BS medium containing PDGF without TH in 1.5% O_2 for 12 days at 37℃. Then the cells were dissociated and harvested. These cells were co-transfected with anti-Hif1α siRNA and pMaxGFP or anti-Hif2α siRNA and pMaxGFP. Cells were re-cultured in PDL-coated slide flasks at clonal-density with BS medium containing PDGF with TH in 1.5% O_2 for 4 days at 37℃. The number of GFP^+ cells in each clone was counted. si-NT; non-target siRNA, si-Hif1a; anti-Hif1α siRNA, si-Hif2a; anti-Hif2α siRNA. P* <0.05, P** < 0.01 (ANOVA with Fisher's LSD test, n = 3).

(**e**) 2,000 of OPCs purified from P7 rat optic nerve were inoculated in PDL-coated T25 flasks. Cells were cultured in BS medium containing PDGF with TH in 3% O_2 or 1.5% O_2 conditions at 37℃. For several flasks in 3% O_2, DMOG (1 mM) was added. After 7 days, the number of cells in each clone was counted. P* < 0.005, P** < 0.05 (ANOVA with Fisher's LSD test, n = 3).

(**f**) 2,000 of OPCs purified from P7 rat optic nerve were inoculated in PDL-coated T25 flasks. Cells were pre-cultured in BS medium containing PDGF with TH in 3% O_2 for 10 days at 37℃. Then, some cells were treated with DMOG (1 mM) for 24 hours. Total RNA was prepared from the cells and a qRT-PCR analysis was carried out. Results

were presented as the relative amount of transcripts to that of the DMOG free culture using comparative ΔΔCt method. *Actb* is an endogenous negative control. *Ldha*, *Pgk1* and *Vegfa* are HIFs-inducible positive control. The P values of these genes are P < 0.001 (ANOVA with Fisher's LSD test, n = 3).

(g) 1,000 of OPCs purified from P7 rat optic nerve were inoculated in PDL-coated slide flasks. Cells were pre-cultured in BS medium containing PDGF with TH in 3% O_2 for 10 days at 37℃. Then, some cells were treated with DMOG (1 mM) for 24 hours. After then, cells were stained with rabbit anti-Runx1 antibodies (*green*) and PI (*red*). Scale bars; 50 μm. Note; in the DMOG treated cells (3% O_2 + DMOG), Runx1 protein increased and colocalized with nucleus (*yellow*).

Supplementary Figure Legend for Figure 7

(a) 2,000 of A2B5$^+$/GC$^-$ OPCs purified from P14 rat optic nerve were inoculated in PDL-coated T25 flasks. Cells were cultured in BS medium containing PDGF with TH (*black bars*) or without TH (*white bars*) in 1.5% O_2 for 13 days at 37℃. After then, the number of cells in each clone was counted and the number of cell divisions was estimated (n = 3). Around 50% of clones contained the cells of which divided less than three times are shown in red square.

(**b**) P7 OPCs and P14 OPCs were purified from rat optic nerve and their total RNA was prepared immediately. qRT-PCR analysis was carried out. The resulting values were normalized to the endogenous control gene, β-actin (*Actb)*. Results of P14 OPCs are presented as the relative expression to those of P7 OPCs using the ΔΔCt methods. P* <0.001, P** < 0.001 (ANOVA with Fisher's LSD test, n = 3).

(c) GC-negative optic nerve cells prepared from P14 rat were sorted with A2B5 monoclonal antibody (*right panel*). These GC$^-$/A2B5$^+$ OPCs were stained for Runx1 and Ki-67 and examined in immunochemistry. The percentages of Runx1 expressing cells in Ki-67$^+$ or Ki-67$^-$ OPCs are shown (*left graph*). *P < 0.01 (unpaired Student's *t*-test, n = 10).

(**d**) Pimonidazole was injected intraperitoneally into P7 rats or P14 rats. 2 hours later, OPCs were purified from the rat optic nerves. These cells were stained for pimonidazole and Ki-67. The percentages of Ki-67$^-$ OPCs that were Pimo$^-$ (*white bars*) or Pimo$^+$ (*black bars*) at P7 and P14 are shown. *P < 0.001 (unpaired Student's *t*-test, n = 4).

Supplementary Figure Legend for Figure 8

(a) 2,000 of OPCs purified from P7 rat cerebral cortex were inoculated in PDL-coated T25 flasks. Cells were cultured in BS medium containing PDGF without TH (-TH; *white bars*) or with TH (+TH; *black bars*), in 5% O_2 or 1.5% O_2 for 11 days at 37℃. Cells were dissociated and the number of cells was counted. $0.01 < *P < 0.02$ (unpaired Student's *t*-test, n = 3).

(**b**) 1,000 of OPCs purified from P7 rat cerebral cortex were inoculated in PDL-coated slide flasks. OPCs were pre-cultured in BS medium containing PDGF without TH (-TH; *white bars*) or with TH (+TH; *black bars*) in 1.5% O_2 for 10 days at 37℃. The cells were treated with BrdU for 20 hours and labeled with anti-BrdU antibody and Hoechst 33342 dye. The percentage of $BrdU^+$ cells was determined. $*P < 0.01$ (unpaired Student's *t*-test, n = 3).

(c) 1,000 of OPCs purified from P7 rat cerebral cortex were inoculated in PDL-coated slide flasks. Cells were cultured in BS medium containing PDGF without TH (-TH; *white bars*) or with TH (+TH; *black bars*) 1.5% O_2 for 11 days at 37℃. They were then stained with the nuclear stain DAPI and a monoclonal anti-GC or A2B5 antibody, and the percentage of the $DAPI^+$ cells that were GC^+ or $A2B5^+$ was determined (unpaired Student's *t*-test, n = 3).

(**d**) 2,000 of OPCs purified from P7 mouse optic nerve were inoculated in PDL-coated T25 flasks. Cells were cultured in BS medium containing PDGF without TH (-TH; *white bars*) or with TH (+TH; *black bars*), in either 3% O_2 or 1% O_2 for 12 days at 37℃. The number of cell divisions was estimated from the cell numbers in each clone (n = 3).

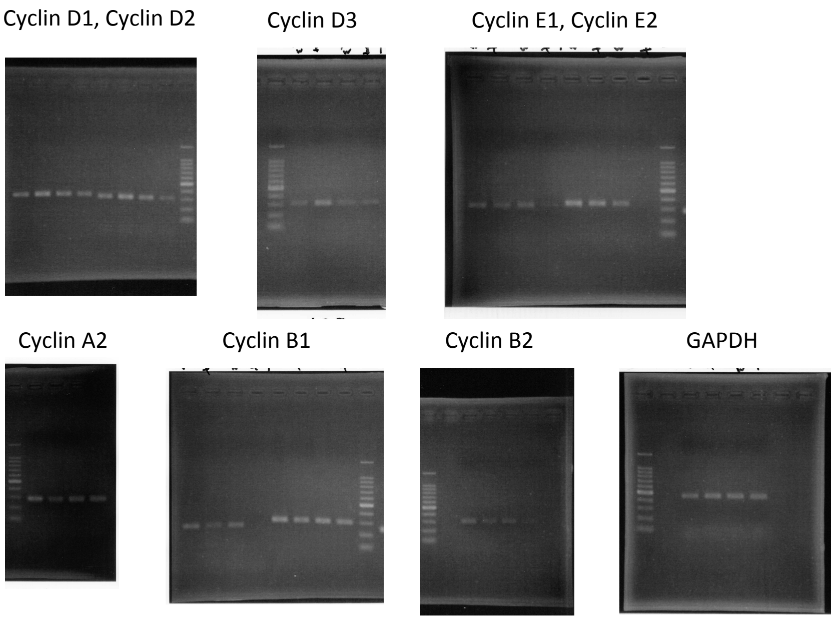

Fig. 4a Original images

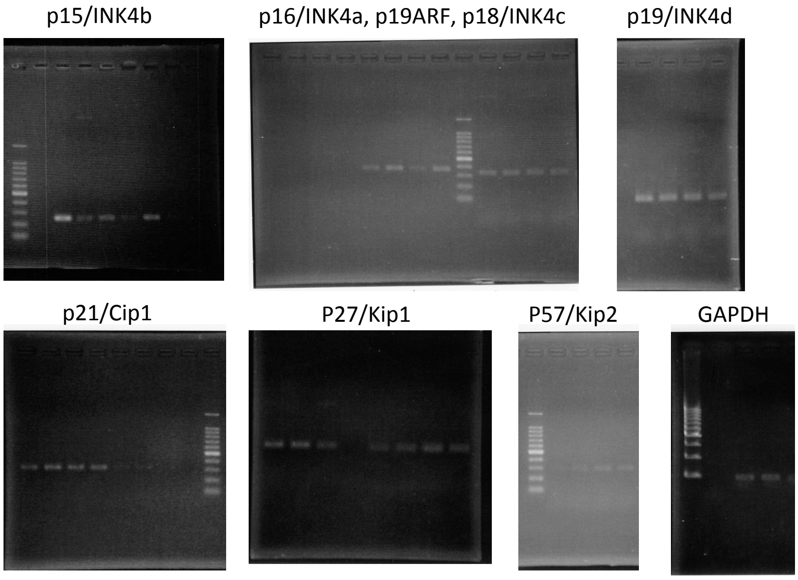

Fig. 4b Original images

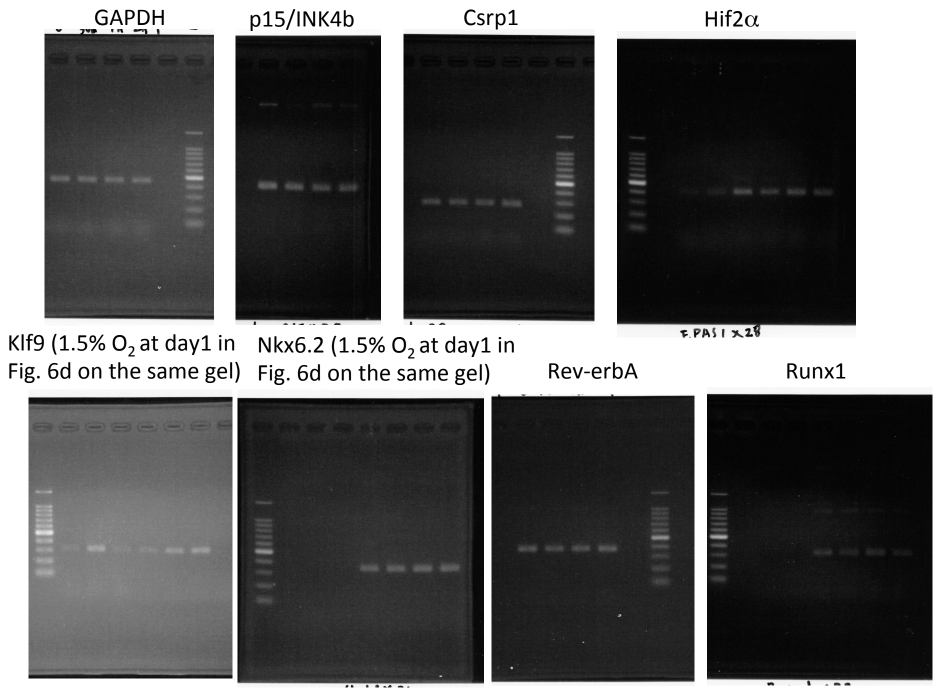

Fig. 5b Original images

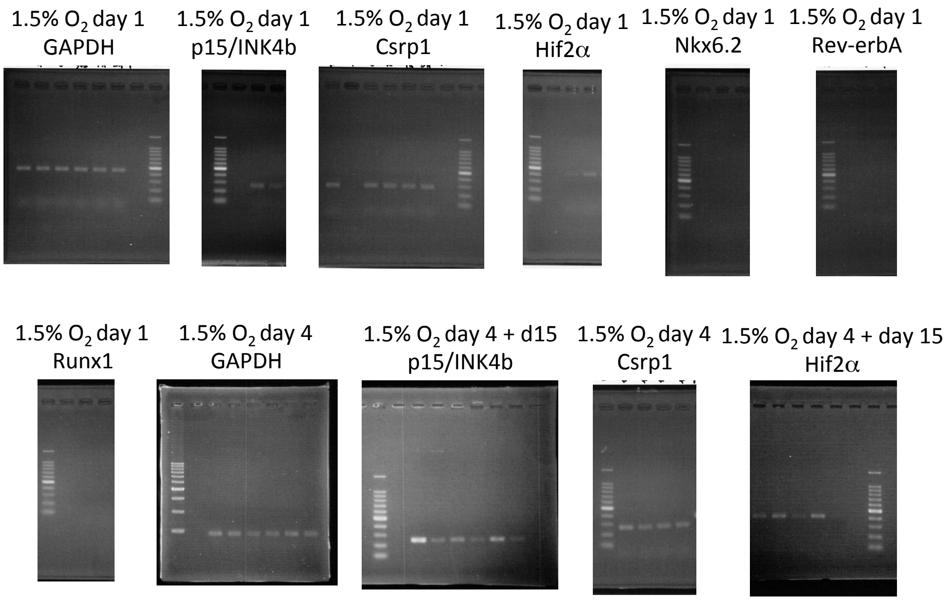

Fig. 5d Original images 1

1.5% O_2 day 4 + day 15
Klf9

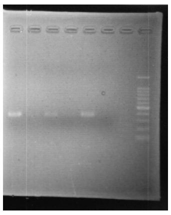

1.5% O_2 day 4
Nkx6.2, Rev-erbA, Runx1

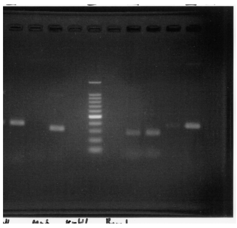

1.5% and 3% O_2 day15
GAPDH

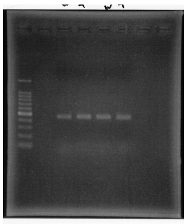

1.5% O_2 day 15
Nkx6.2

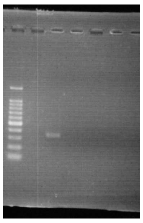

1.5% and 3% O_2 day 15
Csrp1

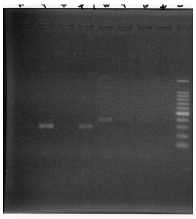

1.5% O_2 day15
Rev-erbA, Runx1

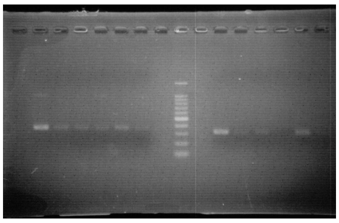

3% O_2 day15
p15/INK4b, Hif2α

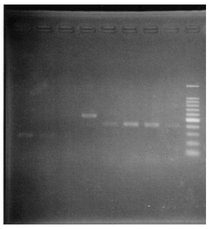

Fig. 5d Original images 2

3% O_2 day 15
Klf9, Nkx6.2, Rev-erbA

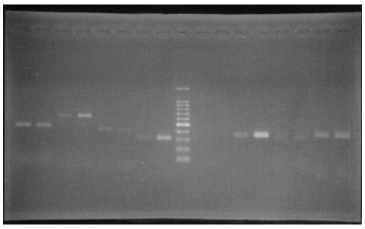

3% O_2 day 15
Runx1

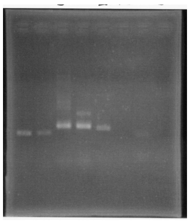

Fig. 5d Original images 3

GAPDH

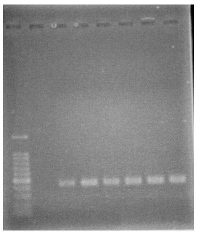

Hif1α

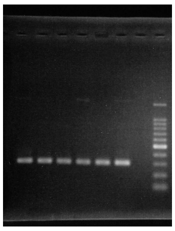

Hif1β

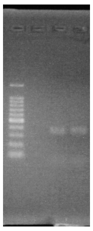

Hif2α

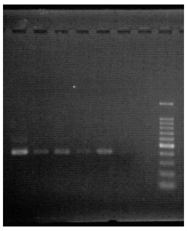

Fig. 6a Original images

Anti-Runx1 antibody Anti-p15/INK4b antibody Anti-GAPDH antibody

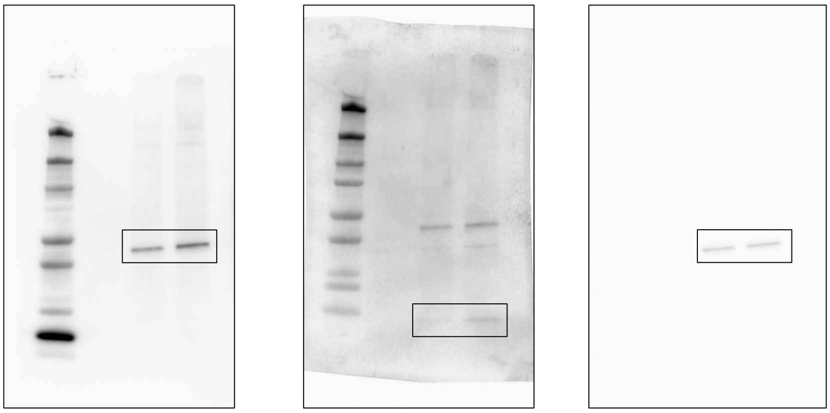

Original data of Supplementary Fig. S3b

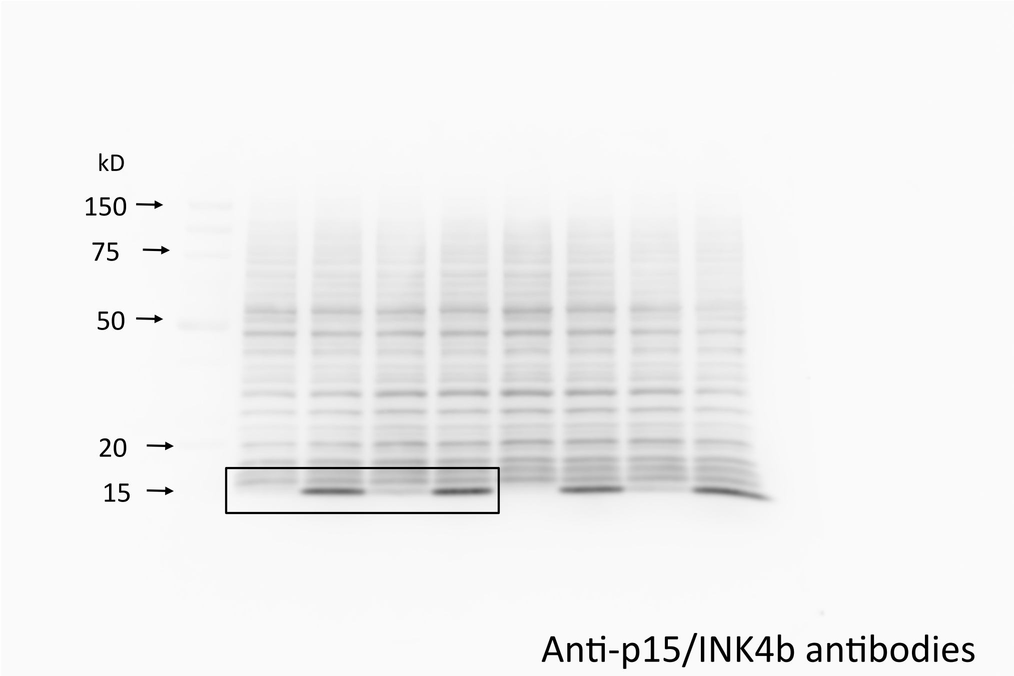

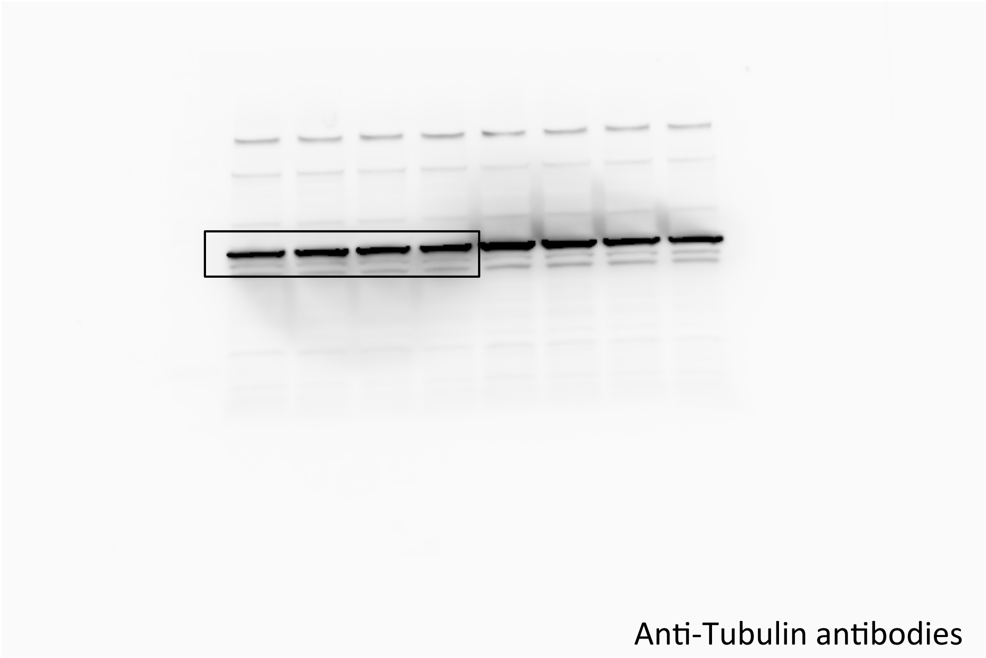

Original image of **Supplementary Fig. S4a.**

Anti-Hif1α antibody

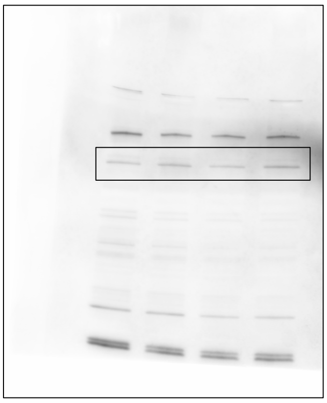

Anti-Tubulin antibody

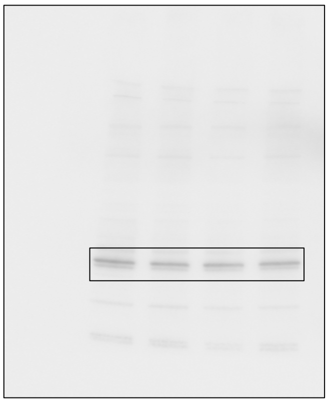

Original data of Supplementary Fig. S4a

Anti-Hif2α antibody

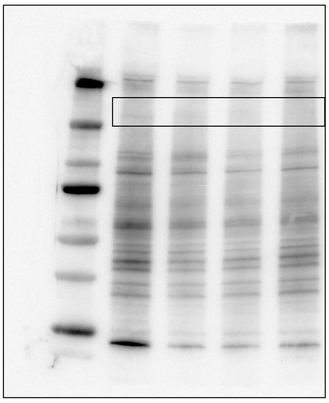

Anti-Tubulin antibody

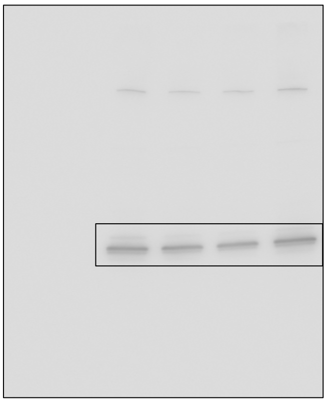

Original data of Supplementary Fig. S4a

Anti-Runx1 antibody

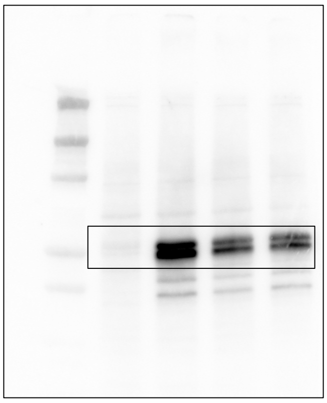

Anti-Tubulin antibody

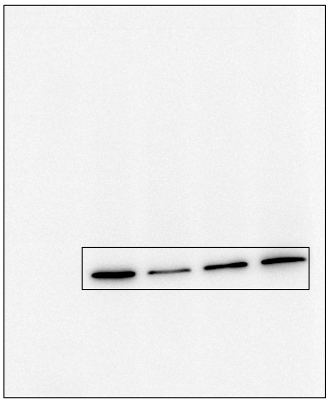

Original data of Supplementary Fig. S4a
